# Multi-responsive biopolymer–lipid hybrid nanocarriers for controlled doxorubicin delivery

**DOI:** 10.1039/d6na00677a

**Published:** 2026-10-09

**Authors:** Öznur Safgöl, Banu Iyisan

**Affiliations:** a Biofunctional Nanomaterials Design (BIND) Laboratory, Institute of Biomedical Engineering, Bogazici University 34684 Istanbul Turkey banu.iyisan@bogazici.edu.tr; b Center for Targeted Therapy Technologies (CT3), Bogazici University 34684 Istanbul Turkey

## Abstract

Hybrid nanocarriers are widely used in anticancer drug delivery because they integrate complementary properties of multiple materials. However, many rely on synthetic surfactants for colloidal stability, which may compromise biocompatibility, while exhibiting limited responsiveness to external stimuli. These challenges highlight the need for more stable and multifunctional delivery platforms. Herein, a surfactant-free biopolymer–lipid hybrid nanocarrier was developed using a lauric acid (LA)/olive oil (OO) lipid core and a bovine serum albumin–hyaluronic acid (BSA : HA) biopolymeric shell. The formulation was optimized using a Taguchi design of experiments (DoE) approach to achieve the desired physicochemical characteristics. The optimized nanoparticles exhibited a sub-200 nm size in water, uniform morphology, and colloidal stability comparable to that of conventionally surfactant-stabilized nanoparticles in various media over 8 weeks. Doxorubicin (DOX) was encapsulated in the optimized HNPs, and *in vitro* release studies under different conditions demonstrated pH-, enzyme-, and short-term temperature-dependent drug release. Compared with conventionally surfactant-stabilized nanoparticles, the developed HNPs exhibited significantly improved cytocompatibility toward mouse fibroblast (L929) cells. Additionally, DOX-loaded HNPs exhibited significant anticancer activity against human breast cancer (MCF-7) cells. Overall, the developed HNP system combines a surfactant-free formulation, enhanced cytocompatibility, and multi-stimuli-responsive drug release, making it a promising platform for controlled cancer drug delivery.

## Abstract 2

Hybrid nanocarriers with an albumin–hyaluronic acid shell and a lauric acid–olive oil core offer a surfactant-free platform for controlled, multi-responsive doxorubicin delivery. Created in BioRender. Ö. Safgöl, (2026) https://BioRender.com/hhh4fgf.

## Graphical abstract

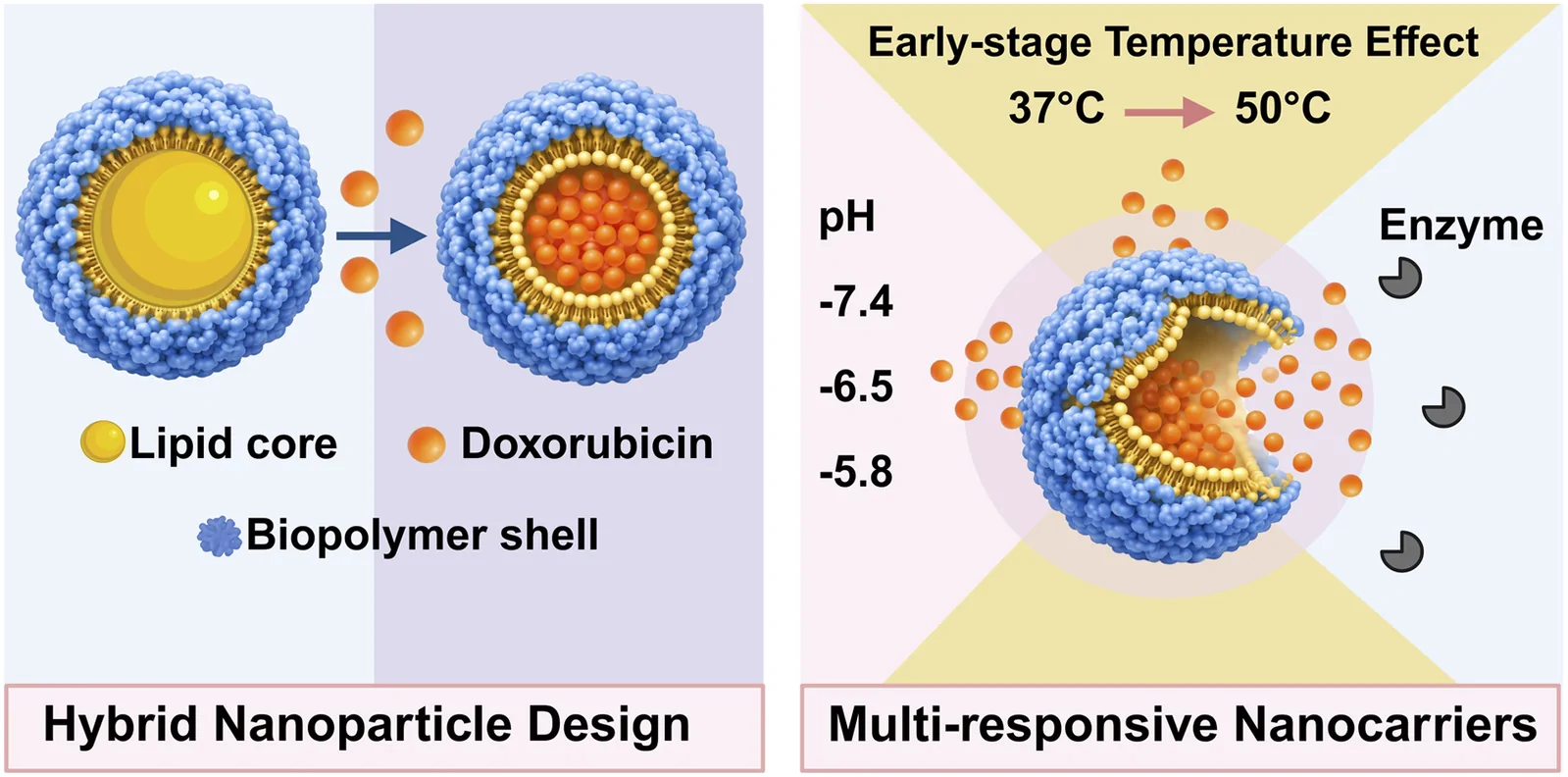

## 1. Introduction

Cancer remains one of the leading causes of morbidity and mortality worldwide.^1^ Its heterogeneous nature and metastatic potential and low tumor selectivity of current treatment approaches present a significant clinical limitation.^2^ Although doxorubicin (DOX) is widely used among chemotherapeutic agents due to its broad-spectrum antitumor activity, its clinical application is limited by dose-dependent cardiotoxicity and systemic side effects.^3,4^ The non-selective biodistribution of DOX leads to damage in healthy tissues and reduces therapeutic efficacy.^5^ Therefore, there is a need for advanced drug delivery systems that can increase the therapeutic index of DOX and provide targeted and controlled release.^6,7^ Nanocarrier-based drug delivery systems have emerged as a promising strategy to improve the pharmacokinetic profile of chemotherapeutics, increase bioavailability, and enable selective tumor accumulation through the enhanced permeability and retention (EPR) effect.^8^ However, conventional nanocarriers fail to retain sufficient long-term colloidal stability and premature drug leakage during systemic circulation.^9^

To address these limitations, polymer–lipid hybrid nanocarrier systems have gained attention due to their biocompatibility and versatile design possibilities.^10^ The lipid core enhances drug-loading capacity, while the polymeric shell improves the system's colloidal stability and ensures controlled release.^11^ However, in most conventional systems, colloidal stability is primarily dependent on synthetic surfactants, which can increase immunogenicity and contribute to increased toxicity in biological environments.^12^ Consequently, biocompatible and functional shell designs free from synthetic surfactants have become increasingly important in recent nanomedicine research.^11,13^ Bovine serum albumin (BSA) is an ideal shell component in such systems due to its amphiphilic nature, biodegradability, and structural similarity to human serum albumin.^14^ In addition to its structural role, BSA-based systems have been observed to interact with tumor-associated albumin-binding pathways, contributing to increased tumor accumulation.^15^

The functionality of BSA-based shells can be enhanced by covalent conjugation with natural polysaccharides.^16^ For instance, Maillard reactions occur between the amino groups of proteins and the reducing carbonyl groups of polysaccharides.^17^ Also, it does not require synthetic cross-linking agents and stands out as a green conjugation approach in the literature.^18^ In this regard, hyaluronic acid (HA) is a natural polysaccharide widely used in drug delivery systems for its high biocompatibility, hydrophilicity, and affinity for CD44 receptors.^19^ This receptor–ligand interaction has been widely used to improve active tumor targeting and cellular uptake.^20^ Therefore, HA is considered a frequently preferred component in the functionalization of protein-based nanocarriers.^21^

In addition to the shell structure, the composition of the lipid core plays a critical role in determining drug loading capacity and physical stability.^22^ Nanostructured lipid carriers (NLCs) composed of solid and liquid lipids have attracted considerable attention due to their high encapsulation efficiency and controlled release properties.^23^ Lauric acid (LA) is widely considered a suitable lipid component owing to its low melting point and phase transition behavior, which may influence drug release upon temperature variation.^24^ Moreover, liquid lipids such as olive oil have been reported to enhance matrix flexibility and improve drug loading capacity.^25^ Together, these lipid components form an appropriate platform for temperature modulated and controlled-release hybrid nanocarrier systems.^24,26^

In hybrid nanocarrier designs, passive drug delivery techniques are frequently insufficient to address the complex and dynamic tumour microenvironment.^27^ Therefore, stimuli-responsive drug delivery systems have gained increasing attention in recent years. Using multi-stimuli-responsive systems capable of responding to multiple biological signals, rather than a single trigger, is an important strategy for controlled drug delivery.^28,29^ The tumor microenvironment and cellular internalization pathways offer significant biological triggers for drug release. Specifically, the difference between physiological pH (≈7.4) and early endosomal/endosomal pH (≈6.5–5.5) enables pH-sensitive systems to respond *via* surface charge and conformational changes.^30^ Additionally, proteolytic enzymes have been investigated as a potential stimulus for enzyme-responsive degradation.^31^ Furthermore, thermal stimuli, either local or externally applied, can modulate release kinetics through phase transitions.^32^ These signals together provide a strong multi-stimulus framework for controlled drug release.^24,33^

This study focuses on the development of a biocompatible and multi-responsive hybrid nanocarrier platform for controlled DOX delivery. The system combines the high drug loading capacity of a lipid core with the colloidal stability and biocompatibility of the BSA : HA-based polymeric shell. This structure also provides a safer delivery matrix by eliminating the need for synthetic stabilizers. Furthermore, the pH- and enzyme-responsive biopolymer shell, together with temperature-dependent behavior associated with the LA-containing core, contributes to the multi-responsive release behavior of the system. While the present study builds on our previous work on BSA–polysaccharide-based biopolymeric systems, which demonstrated improved colloidal stability and reduced toxicity,^34,35^ the current formulation combines a BSA : HA conjugate shell with an LA–OO lipid core and has been systematically optimized and evaluated for controlled, multi-responsive DOX delivery. The overall design and working mechanism of the developed hybrid nanocarrier platform are illustrated in Fig. 1. Briefly, the system was optimized using different formulation parameters, followed by DOX encapsulation, comparative stability evaluation against surfactant-containing systems, investigation of multi-stimuli release behavior, and *in vitro* biological evaluation using MCF-7 breast cancer and L929 mouse fibroblast cells.

**Fig. 1:**
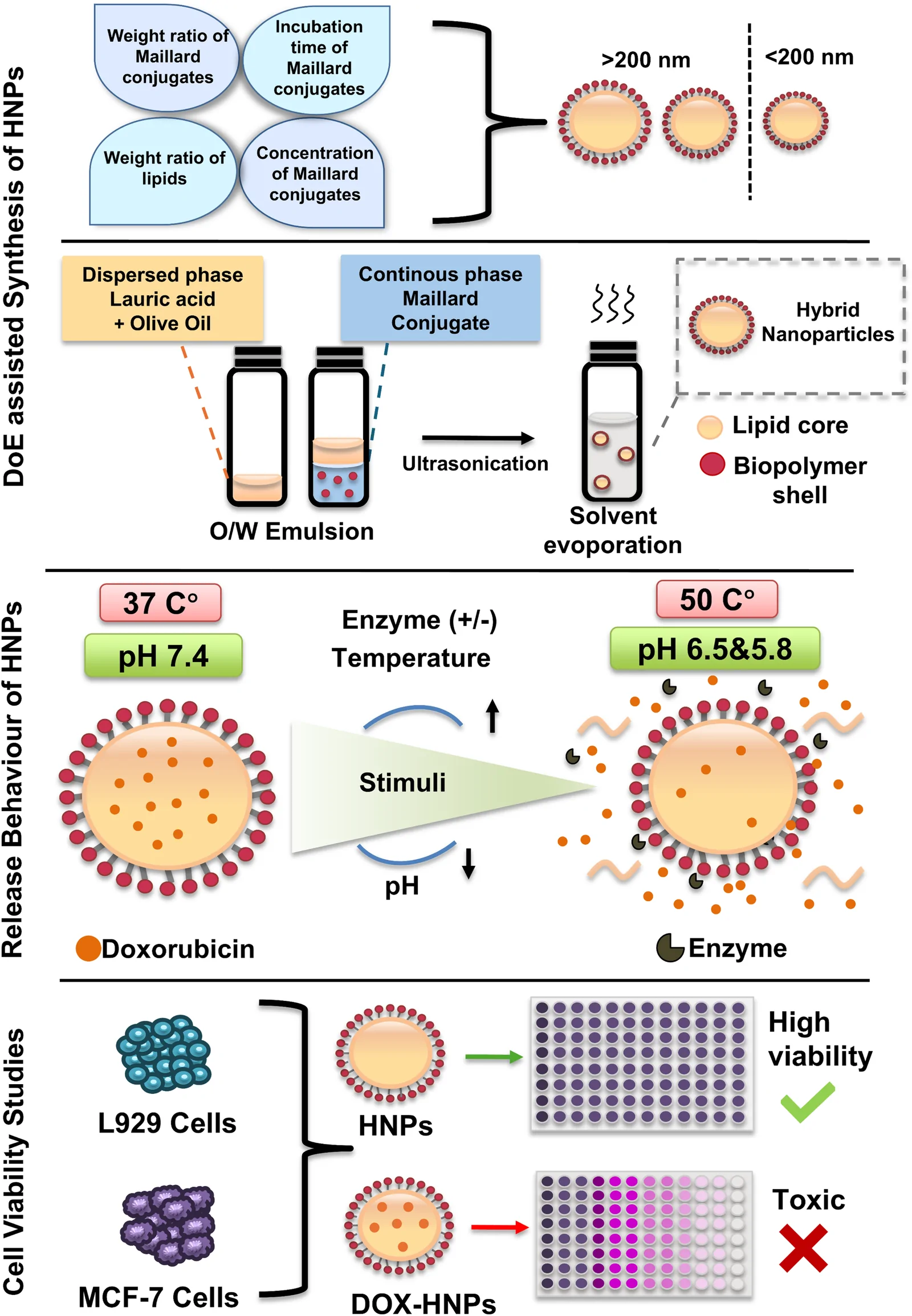
Schematic overview of the study, including the experimental design parameters for synthesizing hybrid nanoparticles (HNPs), encapsulating doxorubicin into optimal HNPs, the release study of the HNPs, and the investigation of HNPs' cytotoxicity on both healthy and cancer cell lines (DoE: Design of Experiments).

## 2. Experimental section

### 2.1. Materials

Bovine serum albumin (BSA, lyophilized powder, ≥98%), olive oil (highly refined, low acidity), lauric acid (natural, ≥98%, FCC, FG), proteinase K from *Tritirachium album* (lyophilized powder, ≥30 units per mg protein), Tween 80, Tween 20, and hydrochloric acid (HCl) were purchased from Sigma-Aldrich. Hyaluronic acid sodium salt (*M*_w_ = 80–100.000 g mol^−1^) and DOX-HCl were purchased from Carbosynth. Ethyl acetate (ACS grade), potassium bromide (KBr), potassium chloride (KCl), monobasic sodium phosphate (NaH_2_PO_4_·H_2_O), dibasic sodium phosphate (Na_2_HPO_4_·7H_2_O), and sodium hydroxide (NaOH) were provided by Acros Organics. Pierce 660 nm Protein Assay Reagent and SDS–PAGE materials, 4× Bolt LDS sample buffer, 10× Bolt reducing agent, 20× Bolt MES SDS running buffer, Bolt Bis–Tris running gels (10%, 10-well), and Invitrogen SimplyBlue safestain (Coomassie Brilliant Blue), were purchased from ThermoFisher Scientific. For cell culture studies, Dulbecco's Modified Eagle's Medium (DMEM), fetal bovine serum (FBS, qualified Brazilian origin), penicillin–streptomycin (10.000 U ml^−1^, 100×), and trypsin–EDTA (0.25% with phenol red) were purchased from Gibco, dimethyl sulfoxide (DMSO, cell culture reagent) was purchased from NutriCulture, thiazolyl blue tetrazolium bromide (MTT Reagent, 98%) was purchased from Thermo Fisher Scientific, and trypan blue was purchased from Hyclone. Dulbecco's calcium- and magnesium-free phosphate-buffered saline (dPBS) was purchased from Gibco (Invitrogen). For drug release of nanoparticles, a dialysis membrane (MWCO 6–8 kDa) was purchased from Spectrum Spectra/Por. Furthermore, filtered pure water was used throughout all experimental procedures.

### 2.2. Methods

#### 2.2.1 Preparation of BSA : HA conjugates

BSA : HA conjugates were prepared *via* the Maillard reaction, a chemical reaction between the amino groups of proteins and the carbonyl groups of reducing carbohydrates upon heating. The conjugation process was carried out according to our previous studies.^34,35^ First, 10 mL of BSA : HA mixtures at different mass ratios (w/w) (Table S1) were prepared in filtered pure water at 1% (w/v). The pH of the mixtures was adjusted to 8 using 0.1 M sodium hydroxide (NaOH), and they were stirred for 16 h. Secondly, the mixtures were lyophilized for 48 h at 0.03 mBar using Labconco 4.5. Then, they were placed in an incubator inside a desiccator containing saturated potassium bromide (KBr) solution to maintain 79% relative humidity over four predetermined time intervals, as seen in Table S1 at 60 °C. Lastly, the obtained conjugates were stored at +4 °C for further characterization and usage.

#### 2.2.2 Characterization of Maillard conjugates

##### 2.2.2.1 Sodium dodecyl sulfate–polyacrylamide gel electrophoresis (SDS–PAGE)

Before applying SDS–PAGE, protein concentrations of BSA : HA conjugates were determined by Thermo Scientific Pierce 660 nm Protein Assay. BSA was used as the standard for the calibration curve as seen in Fig. S1. Absorbance of BSA and dissolved conjugate solutions in pure water at a final concentration of 1 mg mL^−1^ was measured at 660 nm using a TECAN Infinite M Nano microplate reader. Each absorbance measurement was performed in triplicate, and protein concentrations were recorded as seen in Table S1. Based on the Pierce Protein Assay results, the volume of each conjugate containing 2.5 µg of BSA was calculated. As directed by the manufacturer (Invitrogen/Thermo Fisher Scientific), samples were mixed with 2.5 µL of Bolt LDS sample buffer, 1 µL of Bolt sample reducing agent, and ultrapure water. Samples were incubated for 10 min at 70 °C for denaturation. Following denaturation, they were loaded into Bolt 10% Bis–Tris polyacrylamide gels and comigrated using PageRuler Plus Prestained Protein Ladder for 75 min at a constant 100 V. The gel was then stained with SimplyBlue SafeStain while being gently shaken overnight. Ultrapure water was used to rinse it twice for one hour. Lastly, the gel was imaged by scanning using a Bio-Rad Gel Doc XR+ Gel Documentation System.

##### 2.2.2.2 Fourier transform-infrared spectroscopy (FT-IR)

FT-IR was used to examine the chemical characteristics and structures of BSA, HA, and BSA : HA conjugates using an IR Spirit-T spectrometer (Shimadzu). The spectra were collected between the wavenumbers 400 and 4000 cm^−1^.

##### 2.2.2.3 TNBS assay

The 2,4,6-Trinitrobenzene Sulfonic Acid (TNBS) assay is a rapid and sensitive method for determining the degree of conjugation by quantifying free primary amino groups in Maillard conjugates. We performed the TNBS assay as described in the literature.^13^ The BSA : HA conjugates with varying weight ratios and incubation times were dissolved at a concentration of 0.2 mg mL^−1^ in reaction buffer (0.1 M sodium bicarbonate, pH 8.5). To produce the standard calibration curve, BSA solutions were diluted in reaction buffer from 0 to 200 µg mL^−1^, as shown in Fig. S2.

Subsequently, 0.25 mL of freshly prepared 0.01% (w/v) TNBS solution was added to 0.5 mL of each sample or standard solution. The TNBS solution was freshly prepared and diluted immediately before each use. The samples were mixed at 1000 rpm for 5 min using a thermoshaker. Then, the mixtures were incubated at 37 °C for 2 h. Following incubation, 0.25 mL of 10% (w/v) SDS solution prepared in distilled water and 0.125 mL of 1 M HCl were added to each sample to terminate the reaction. The absorbance was then measured at 335 nm using a TECAN Infinite M Nano microplate reader, allowing quantification of the degree of conjugation (%), (DC), using eqn (1).1
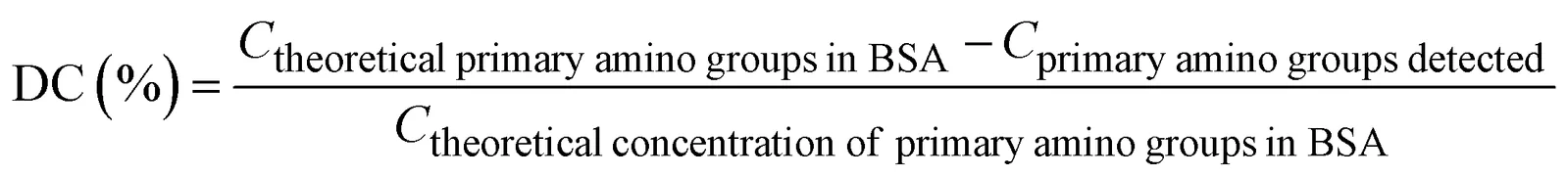
where the theoretical concentration of primary amino groups represents the total concentration of primary amino groups initially present in the added BSA and the concentration of free primary amino groups represents the concentration measured in the sample after the conjugation reaction.

#### 2.2.3 Synthesis of hybrid nanoparticles (HNPs)

The synthesis of HNPs was performed *via* a direct miniemulsion-solvent evaporation method, as previously outlined in our study,^34^ with slight modifications introduced in the present work. First, BSA–HA conjugates formed at different incubation times were dissolved in 10 mL of pure water for each of four concentration levels to obtain the aqueous (continuous) phase. Additionally, a mixture of olive oil (OO) and lauric acid (LA) was dissolved in 2 mL of ethyl acetate to obtain the dispersed phase. Subsequently, the two phases were combined and mixed at 1000 rpm for 10 seconds to form a macroemulsion, and ultrasonication (Branson 550 Sonifer) was performed under an ice bath at 90% amplitude (15-second pulse and 5-second pause) for a total of 5 min. Lastly, the ethyl acetate was evaporated at ambient temperature while being stirred at 250 rpm for 16 hours. Four factors, conjugate weight ratio (BSA : HA, w/w) (*A*), conjugate incubation time (h) (*B*), lipid weight ratio (LA : OO, w/w) (*C*), and shell concentration (mg mL^−1^) (*D*), were analyzed according to the DOE (Taguchi design) with four levels *via* the synthesis of nanoparticles.

#### 2.2.4 Characterization of hybrid nanoparticles

The average hydrodynamic diameter (nm), polydispersity index (PDI), and zeta potential (*ζ*-potential, mV) of nanoparticles were measured by dynamic light scattering (DLS) and electrophoretic light scattering (ELS) at 25 °C and an angle of 90° by using a Zetasizer Lab (Malvern Instruments, U.K.). For each measurement, 50 µL of nanoparticle dispersion was diluted with 950 µL of pure water (1 : 20, v/v). For *ζ*-potential measurements, a 1 mM KCl solution was used at the same concentration to provide a standardized ionic environment. The solid content of the nanoparticles was determined gravimetrically by drying 100 µL of dispersion in triplicate. For the confirmation of the morphology of the HNPs, scanning transmission electron microscopy (STEM) was performed using a Quattro S instrument (Thermo Fisher Scientific). A drop of aqueous HNP dispersion was placed on a 300-mesh carbon-coated copper grid and left to dry. Then, the STEM was operated at an acceleration voltage of 30.00 kV. FT-IR was used to analyze the chemical structures of lyophilized nanoparticles by using an IR Spirit-T spectrometer from Shimadzu. The spectra were collected between the wavenumbers 1000 and 4000 cm^−1^.

#### 2.2.5 Design of experiments

The hydrodynamic diameter of nanoparticles as a response value was investigated *via* the design of experiments. With selected parameters and levels, the samples were analyzed using a Taguchi experimental design. A total of 16 formulations with 4 parameters (A, B, C, and D) at 4 levels were subjected to analysis. Each formulation was synthesized in three independent batches, and each batch was analyzed in triplicate. Mean effects and ANOVA analysis were performed using the free trial of Minitab Statistical Software 2022. The nanoparticles, with their optimal parameters determined, were synthesized and used for further characterization.

#### 2.2.6 Synthesis of lipid nanoparticles using conventional surfactants as a control experiment

To evaluate the stabilization effect of BSA : HA conjugates, control nanoparticles (CNPs) were synthesized using conventional surfactants without using a biopolymer shell. First, the nonionic surfactant Tween 20 was added to 10 mL of pure water to form the aqueous (continuous) phase at a concentration of 1% (v/v) for CNP1 and 1 mg mL^−1^ for CNP2. LA : OO (1.5, w/w) was then dissolved in 2 mL of ethyl acetate to create the dispersed phase, according to the optimal lipid ratio selected in the design of the experiment. Subsequently, CNPs were synthesized using the same method as the miniemulsion-solvent evaporation procedure described previously. Characterization of CNPs was performed similarly to that of the HNPs.

#### 2.2.7 pH-dependent zeta potential and size analysis

The pH-dependent zeta potential (mV) and hydrodynamic diameter (nm) measurements of BSA, HA, BSA : HA conjugates, HNPs, and CNPs were performed using dynamic light scattering and electrophoretic light scattering techniques (Zetasizer Lab, Malvern, UK). The pH adjustments were carried out with 0.1 mol per L HCl and 0.1 mol per L NaOH solutions. Experiments were performed between pH 2 and 12.

#### 2.2.8 Colloidal stability analysis

Long-term stability monitoring of optimal HNP and CNP formulations was performed through DLS and ELS measurements at predetermined time intervals. After synthesis, stock dispersions of nanoparticles were diluted 1 : 20 (v/v) with different buffers and stored at 4 °C for hydrodynamic size (nm), PDI, and zeta potential (mV) measurements.

#### 2.2.9 Doxorubicin encapsulation and encapsulation efficiency

DOX-HCl was encapsulated into the HNP formulation optimized by DoE analysis using the same synthesis method. First, 5 mg of DOX-HCl was dissolved in 1 mL of ethyl acetate and mixed with a lipid phase composed of LA : OO (1.5, w/w), also dissolved in 1 mL of ethyl acetate. The rest of the synthesis was carried out following the same procedure as for the HNPs. Then, the formed nanoparticles were purified from nonencapsulated DOX-HCl by centrifugation at 20 000 rpm for 1 h at room temperature. The centrifugation process was repeated twice to ensure the purity of the miniemulsion. To calculate the encapsulation efficiency (EE%) of DOX-loaded-HNPs (DOX-HNPs), supernatants were collected after centrifugation, and absorbance values at 480 nm were measured using Ultraviolet-Visible (UV-vis) spectroscopy (Tecan M Nano). The encapsulation efficiency of DOX-HNPs was calculated by using the DOX-HCl calibration curve shown in Fig. S3 and eqn (2). The drug loading content (DLC) was calculated using eqn (3), considering the mass of the nanoparticles.2
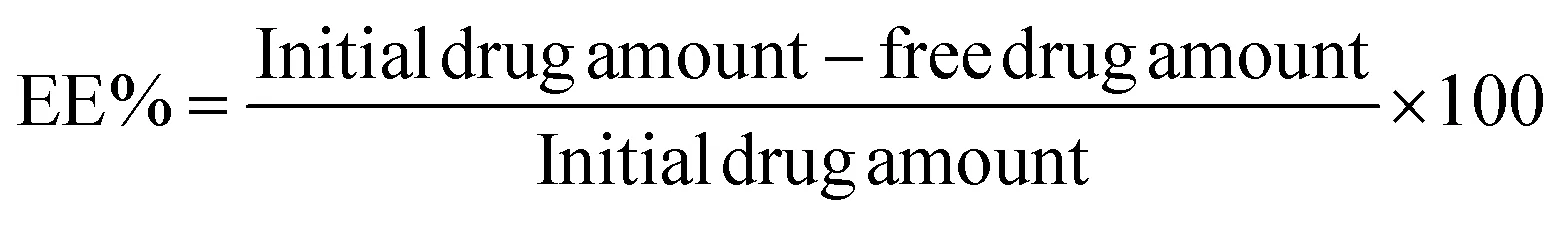
3
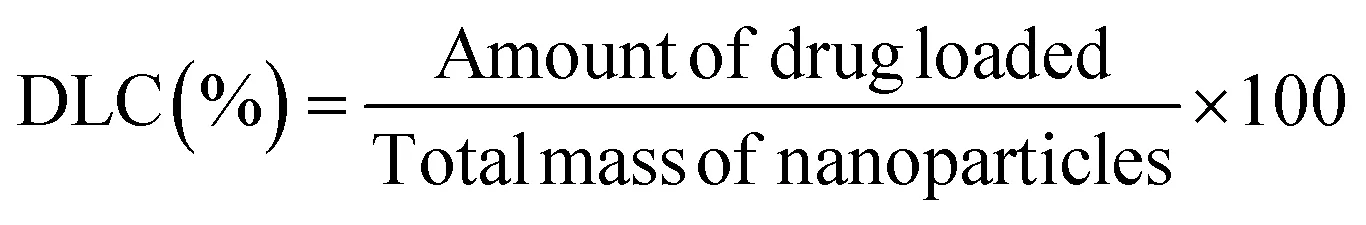


#### 2.2.10 *In vitro* drug release study

The *in vitro* release profile of DOX was evaluated using the dialysis membrane method by varying the pH, temperature, and enzymatic conditions. Following the final centrifugation step described above, nanoparticle dispersions were redispersed in phosphate-buffered saline (PBS, pH 7.4) or potassium phosphate (KPHO, pH 6.5 and 5.8) buffers, and then 1 mL of nanoparticle dispersion was loaded into dialysis membranes (MWCO 6–8 kDa). For each condition, the release of DOX from HNPs was investigated at 37 °C and 50 °C (above the melting point of LA) under magnetic stirring at 200 rpm in triplicate. For enzymatically triggered release, DOX-HNPs under the three pH conditions were incubated with proteinase K from *Tritirachium album* (8 U mg^−1^, to be equal to enzyme/protein ratio: 1/2) and then drug release was performed at 37 °C. Dialysis bags were immersed in beakers containing 100 mL of different buffer solutions (at pH 7.4, 6.5, and 5.8) supplemented with 0.3% Tween 80 (TW80). Samples (600 µL) were withdrawn at predetermined time intervals until 96 h. After each sampling, an equal volume of fresh buffer containing 0.3% Tween 80 was added to maintain sink conditions. The amount of released DOX was quantified by measuring absorbance at 480 nm using a UV-vis spectrophotometer (Infinite M Nano, TECAN, USA). All measurements were performed in triplicate (*n* = 3), and DOX concentrations were calculated using the calibration curve at pH 7.4, pH 6.5, and pH 5.8. (Fig. S4). The release of free DOX at the same concentration (337.8 ± 20.1 µg mL^−1^) from DOX-HNPs under the same conditions was investigated to understand the controlled-release behavior of HNPs better. Furthermore, based on eqn (4), the cumulative drug release under each condition was calculated.4
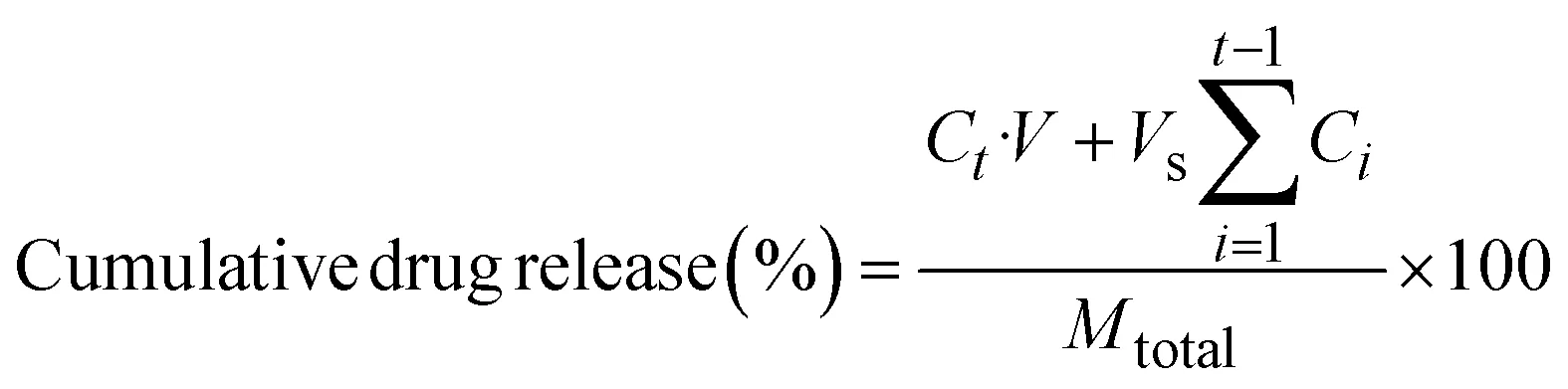
where *C*_*t*_ is the DOX concentration at time (*t*) (mg mL^−1^), *C*_*i*_ is the DOX concentration measured at each preceding sampling point (mg mL^−1^), *V* is the total volume of release medium (100 mL), *V*_s_ is the sampling volume (0.6 mL), and *M*_total_ is the initial amount of DOX encapsulated in the nanoparticles (mg).

#### 2.2.11 Release kinetics

The drug release kinetics from DOX-HNPs were evaluated under different pH (5.8, 6.5, and 7.4), temperature (37 °C and 50 °C), and enzyme presence/absence conditions. Zero-order, first-order, Higuchi,^36^ and Korsmeyer–Peppas kinetic models^37^ were applied to evaluate the release mechanism, as shown in Table 1. Model fit was assessed using linear regression analysis, and the coefficient of determination (*R*^2^) was calculated for each model. The diffusion exponent (*n*), which defines the release mechanism in the Korsmeyer–Peppas model, was also calculated. All kinetic analyses were performed using MATLAB R2023b (MathWorks, USA), and graphical representations were prepared using OriginPro 2026 (OriginLab Corporation, USA) software. The most suitable kinetic model was determined based on the *R*^2^ values.

**Table 1:** Kinetic drug release model equations^a^

Kinetic models	Model equations
Zero-order model	*Q*_*t*_ = *Q*_0_ + *K*_0_*t*
First-order model	log *Q*_*t*_ = log *Q*_0_ − *K*_1_*t*/2.303
Higuchi model	*Q*_*t*_ = *K*_H_*t*^1/2^
Korsmeyer–Peppas model	*Q*_*t*_/*Q*_∞_ = *K*_KP_*t*^*n*^

a *Q*_*t*_ is the cumulative release at time *t*; *Q*_0_ refers to the initial drug concentration in the solution; *K*_0_, *K*_1_, and *K*_H_ correspond to the release rate constants for zero-order, first-order, and Higuchi models, respectively; *Q*_*t*_/*Q*_∞_ is the fraction of drug release at a given time *t*; *K*_KP_ is the release rate constant of the Korsmeyer–Peppas model; and *n* is the release exponent.

#### 2.2.12 Cell culture

For cell culture studies, MCF-7 human breast cancer cells (ATCC, HTB-22) and L929 mouse fibroblast cells (ATCC, CCL-1) were used. Both cell lines were cultured in Dulbecco's Modified Eagle's Medium (DMEM, high glucose, Gibco), which was supplemented with 10% (v/v) fetal bovine serum (FBS, Gibco) and 1% penicillin/streptomycin (Gibco). The cell lines were incubated at 37 °C in a CO_2_-enriched atmosphere (Nüve EC160, Türkiye) with 95% humidity. Under standard incubation conditions, cells were trypsinized for passage using 0.25% trypsin–EDTA (Gibco) for 5 min. Complete growth medium was added to inhibit the enzymatic process, and the cell suspension was centrifuged for 5 min at 1000 rpm. The cell pellet was resuspended in fresh medium after the supernatant was removed. An automated cell counter (BIO-RAD TC20) was used to assess viable cell density using trypan blue staining.

#### 2.2.13 Cell viability assay

Cell viability was assessed using the 3-(4,5-dimethylthiazol-2-yl)-2,5-diphenyltetrazolium bromide (MTT) assay and the effects of optimal HNPs, DOX-HNPs and CNPs were compared. A 96-well plate was seeded with 10.000 cells per well in 100 µL of complete growth media. Following a 24-hour seeding period, cells were cultured for another 24 hours with a 1 : 10 v/v diluted fresh complete growth medium that contained nanoparticle dispersions at different concentrations (5–800 µg mL^−1^). The plate included experimental wells, a medium-only negative control, and untreated control cells, all of which were evaluated in triplicate (*n* = 3). Following 24 and 48 hours of incubation, the medium was removed, and 100 µL of dPBS was used to wash the wells. After that, 100 µl of 10% MTT solution was added to each well, and the plate was incubated for 3.5 hours at 37 °C with 5% CO_2_. To dissolve the formazan crystals formed by metabolically active cells, 100 µl of DMSO was added after removing the MTT-containing medium. The plate was then shaken orbitally for around 15 min to ensure that the formazan crystals were completely dissolved. Finally, absorbance (*A*) was measured at 570 nm using a TECAN Infinite M Nano microplate reader, allowing quantification of cell viability using eqn (5).5
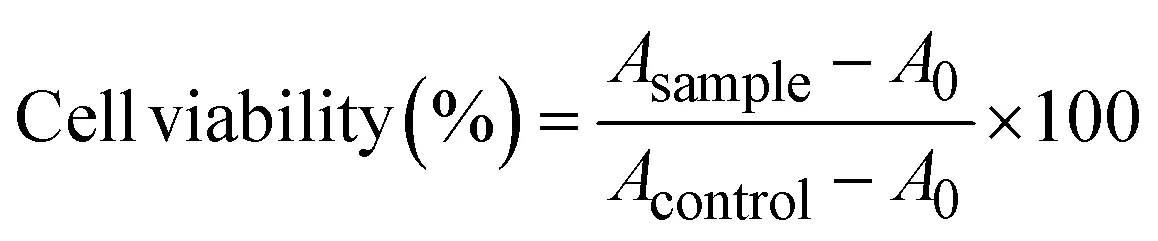


#### 2.2.14 Statistical analysis

Cytocompatibility assays were performed in triplicate. Statistical analyses were conducted using two-way ANOVA. For comparisons between HNP and DOX-HNP groups, multiple comparisons were performed using Sidak's *post hoc* test. For comparisons among HNP, CNP1, and CNP2 groups, Tukey's *post hoc* test was applied to assess differences between all pairwise group combinations. A *p*-value of ≤0.05 was considered statistically significant within a 95% confidence interval. All results were presented as the mean ± standard deviation (SD).

## 3. Results and discussion

### 3.1. Preparation and characterization of BSA : HA conjugates

The amino groups of BSA and the carbonyl groups of HA were covalently conjugated *via* the Maillard reaction, following our previously established methodology.^34,35^ The biopolymer mixture (10 mg mL^−1^) was lyophilized and incubated at 60 °C to induce the Maillard reaction for several incubation times. In this study, as shown in Table S1, a total of 16 different Maillard conjugates were obtained by varying the molecular weight ratios of BSA : HA (w/w, 1 : 1, 3 : 1, 5 : 1, and 6 : 1) and the incubation times of BSA : HA (24, 48, 72, and 96 h). The changed parameters were preserved at four levels for the Taguchi experimental analysis. Protein amount determination was performed using the Pierce 660 nm Protein Assay to find the protein concentrations of each conjugate formed (Table S1). The determined concentrations were used in SDS–PAGE characterization to support the presence of BSA in Maillard conjugates (Fig. S5). According to the SDS–PAGE results, both BSA and BSA : HA conjugates showed a band at around 70 kDa, corresponding to the 66.5 kDa molecular weight of native BSA.^38^ To further quantify the DC of BSA with HA, we determined the DC value (%) using the TNBS assay. As shown in Table S2, the DC ranged from 21.4 ± 1.7% to 60.3 ± 2.1% across the BSA : HA ratios and incubation times investigated. The highest degree of conjugation was obtained for the 6 : 1 BSA : HA ratio after 96 h of incubation (60.3 ± 2.1%). Overall, DC generally increased with increasing BSA : HA ratio, with the 6 : 1 formulation exhibiting the highest DC at all incubation times. This trend may be associated with the greater availability of primary amino groups resulting from the increased BSA content during the Maillard reaction, as covalent BSA : HA conjugates formed through the Maillard reaction have previously been reported.^39^ Furthermore, the DC generally increased with prolonged incubation, although the extent of this increase depended on the BSA : HA ratio. The higher BSA : HA ratios (3 : 1, 5 : 1, and 6 : 1) continued to show increased DC up to 96 h, whereas the 1 : 1 formulation exhibited an apparent plateau after 48 h (33.7 ± 5.5%). This observation suggests that extending the incubation time beyond 48 h did not substantially increase the measured DC at the 1 : 1 ratio under the investigated reaction conditions. The use of amino-group consumption as an indicator of BSA : HA conjugation is also consistent with previous characterization of BSA : HA conjugates using the TNBS assay, where substantial consumption of amino groups was observed following conjugate formation.^40^ These findings provided a quantitative assessment of BSA : HA conjugation under the investigated reaction conditions. The resulting conjugates were subsequently evaluated in nanoparticle formulations to investigate their effects on particle size and identify the formulation condition providing the desired physicochemical characteristics.

FT-IR analysis was employed to investigate the structural characteristics of the Maillard reaction and to compare them with those of pure BSA and HA (Fig. 2). Pure BSA exhibited two characteristic peaks at approximately 1650 and 1520 cm^−1^, corresponding to the amide I and amide II stretching vibrations, respectively.^41^ In contrast, the FT-IR spectrum of HA showed a broad O–H stretching band between 3200 and 3400 cm^−1^, asymmetric stretching vibrations of amide I and/or carboxylate (–COO^−^) groups at around 1600–1650 cm^−1^, and C–O–C/C–O stretching vibrations in the 1030–1150 cm^−1^ region.^42^ The spectra of the Maillard conjugates contained the characteristic peaks of both BSA and HA, regardless of incubation time or the BSA : HA weight ratio. In particular, the amide I (1650 cm^−1^), amide II (1520 cm^−1^), and broad O–H stretching band (3290 cm^−1^) were consistently detected in all conjugate samples. The simultaneous presence of these characteristic bands indicates the coexistence of BSA and HA within the conjugates. This is consistent with the successful formation of Maillard conjugates *via* the Maillard reaction.^43^

**Fig. 2:**
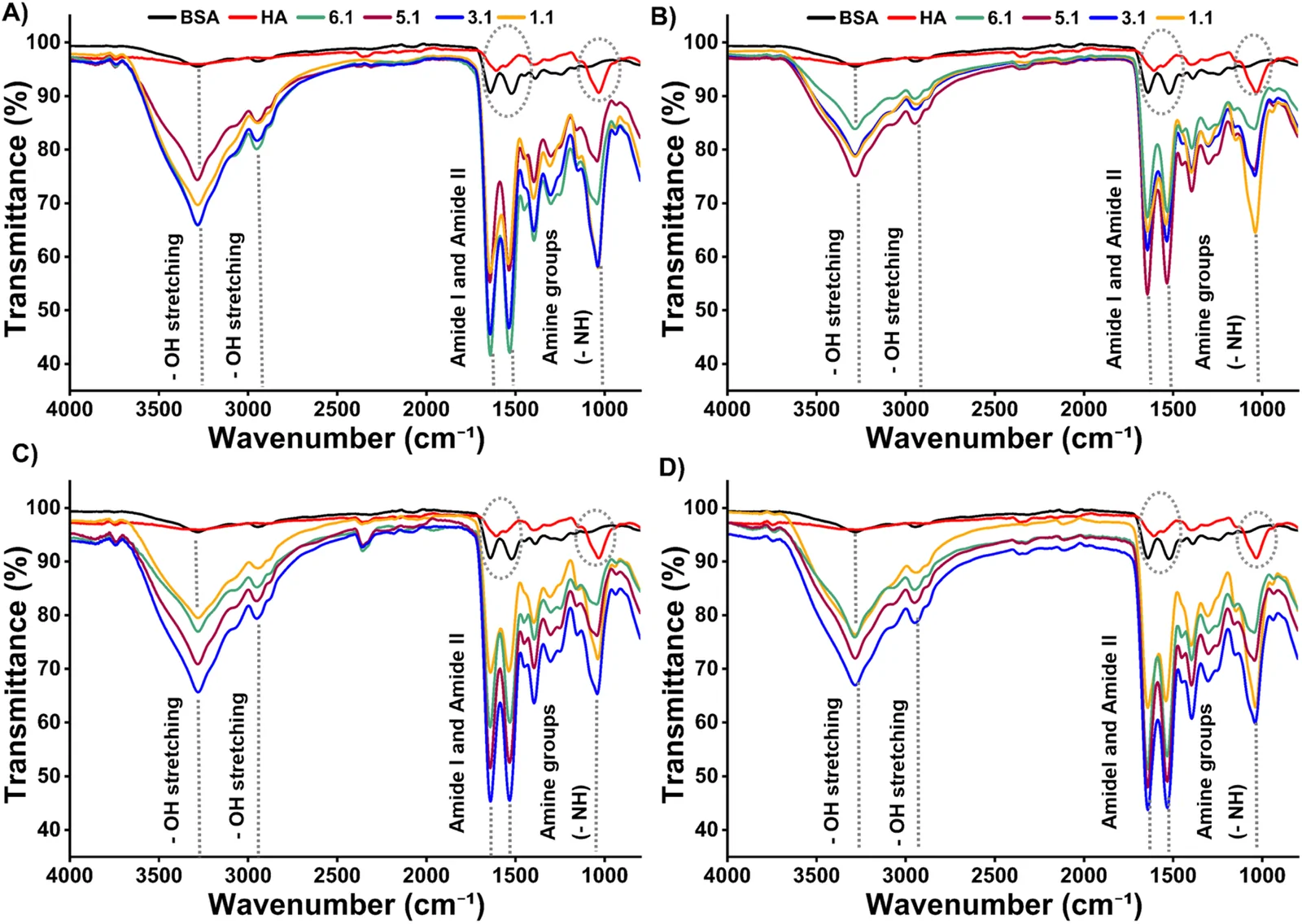
FT-IR spectrum of BSA (black), HA (red), and BSA : HA complexes with different weight ratios (6 : 1, 5 : 1, 3 : 1, and 1 : 1) and incubation times: (A) 24 h, (B) 48 h, (C) 72 h, and (D) 96 h.

### 3.2. The effect of process parameters on size using Taguchi experimental design

In the synthesis of HNPs, four key parameters were identified as potential determinants of the preparation conditions: the conjugate weight ratio (BSA : HA, w/w), conjugate incubation time (h), shell conjugate concentration (mg mL^−1^), and core lipid ratio (LA : OO, w/w). To systematically investigate their effects on particle size, a Taguchi orthogonal array design (L16, 4 factors-4 levels) was employed. The experimental levels are summarized in Table 2. All HNP formulations were prepared in triplicate using the miniemulsion solvent evaporation method,^44^ followed by comprehensive physicochemical characterization (Table 3).

**Table 2:** Experimental factors and levels for the L16 orthogonal Taguchi design

Experimental parameters	Level 1	Level 2	Level 3	Level 4
(A) Conjugate weight ratio (BSA : HA, w/w)	1 : 1	3 : 1	5 : 1	6 : 1
(B) Conjugate incubation time (h)	24	48	72	96
(C) Conjugate concentration (mg mL^−1^)	0.5	1	2	3
(D) Lipid ratio (LA : OO, (w/w))	0.66	0.33	1.5	3

**Table 3:** Experimental parameters (A–D) and the corresponding physicochemical characterization of hybrid nanoparticles (A): conjugate weight ratio (BSA : HA, w/w), (B): conjugate incubation time (h), (C): conjugate concentration (mg mL^−1^), and (D): lipid ratio (LA : OO, w/w). Data are presented as mean ± SD (*n* = 3)

Sample code	Experimental parameters				Physicochemical characterization results		
	A	B	C	D	Size average (nm)	PDI average	Zeta potential average (mV)
HNP1	1	1	1	1	332.4 ± 41.3	0.19 ± 0.03	−48.4 ± 0.8
HNP2	1	2	2	2	306.0 ± 41.1	0.11 ± 0.01	−35.7 ± 11.3
HNP3	1	3	3	3	301.3 ± 9.5	0.08 ± 0.02	−43.2 ± 1.5
HNP4	1	4	4	4	266.6 ± 21.7	0.08 ± 0.02	−31.1 ± 1.8
HNP5	2	1	3	2	305.1 ± 19.1	0.11 ± 0.04	−51.2 ± 0.9
HNP6	2	2	4	1	298.2 ± 14.8	0.15 ± 0.01	−39.9 ± 0.9
HNP7	2	3	1	4	245.6 ± 26.9	0.09 ± 0.03	−34.3 ± 8.6
HNP8	2	4	2	3	200.3 ± 14.1	0.09 ± 0.03	−33.5 ± 3.8
HNP9	3	1	4	3	220.9 ± 14.9	0.11 ± 0.02	−42.9 ± 12.8
HNP10	3	2	3	4	237.5 ± 28.1	0.10 ± 0.00	−46.3 ± 3.9
HNP11	3	3	2	1	271.7 ± 8.8	0.13 ± 0.02	−44.4 ± 4.0
HNP12	3	4	1	2	256.2 ± 9.5	0.14 ± 0.03	−39.3 ± 15.7
HNP13	4	1	2	4	238.4 ± 12.5	0.11 ± 0.04	−50.3 ± 2.0
HNP14	4	2	1	3	242.5 ± 5.7	0.10 ± 0.00	−48.1 ± 1.7
HNP15	4	3	4	2	261.9 ± 17.7	0.12 ± 0.03	−44.2 ± 8.0
HNP16	4	4	2	1	211.4 ± 27.0	0.08 ± 0.01	−46.4 ± 2.1

The colloidal stability of lipid nanoparticles is strongly influenced by the formulation composition, which affects particle size, size distribution, and aggregation behavior. Accordingly, particle size was selected as the primary response variable in this study, and the effects of process parameters were systematically evaluated. PDI and zeta potential were also monitored as complementary indicators of colloidal characteristics. Since the PDI values of all formulations remained below 0.20 and the zeta potential values were consistently negative and generally more negative than −30 mV, with relatively comparable values among the formulations, these parameters did not provide sufficient differentiation for formulation optimization. Therefore, particle size was used as the primary optimization response, while PDI and zeta potential were considered supporting quality attributes.

For quantitative evaluation, the experimental data were analyzed using Minitab Software 22 based on the Taguchi experimental design. The signal-to-noise (S/N) ratio was used to assess the experimental responses. Since the aim of this study was to minimize particle size, the “smaller-is-better” criterion was selected, and the S/N ratio was calculated using eqn (6).^45^6
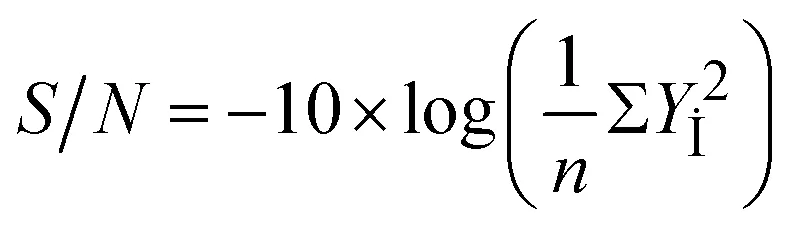
Here, *n* represents the number of tests, while *Y* denotes the response factor.

The main effect analysis indicated that increasing both the conjugate weight ratio and incubation time improved particle size outcome (Fig. 3).

**Fig. 3:**
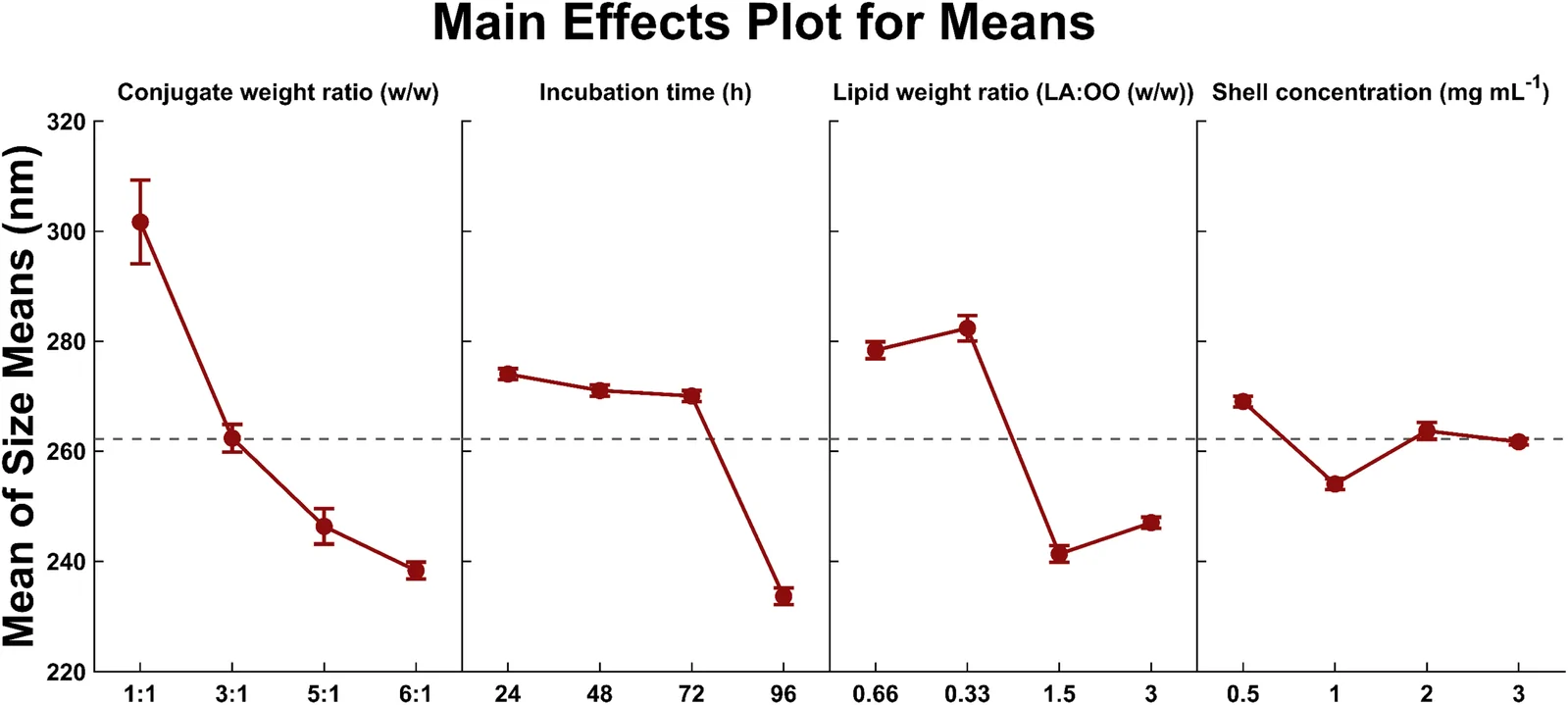
The main effects plots of selected factors on particle size (nm).

Table S3 summarizes the Taguchi analysis results for hydrodynamic particle size. The conjugate weight ratio had the greatest effect on particle size, as indicated by the highest delta value (63.0). Increasing the conjugate weight ratio resulted in a gradual decrease in particle size, suggesting that higher BSA content enhances interfacial stabilization during nanoparticle formation.

The lipid weight ratio (LA : OO, w/w) was the second most influential factor, with a delta value of 40.6. Changes in the core lipid composition may affect particle size by altering lipid phase viscosity and droplet formation during nanoparticle synthesis. Incubation time had a moderate effect, with longer incubation generally resulting in smaller nanoparticles, possibly due to structural rearrangements within the BSA : HA conjugate. In contrast, shell concentration showed the lowest delta value (15.1), indicating a minimal effect on particle size within the investigated range. Therefore, the shell concentration previously optimized in our earlier study was retained for the subsequent experiments. Although the ANOVA (Table S4) results were not statistically significant (*p* > 0.05), the low residual error (*η*^2^ = 0.092) indicates a reliable experimental model. The Taguchi analysis consistently identified the conjugate weight ratio as the most influential parameter governing nanoparticle size.

Based on these findings, the optimal formulation was determined as follows: a conjugate weight ratio of 6 : 1 (BSA : HA, w/w), conjugate incubation time of 96 h, lipid ratio of 1.5 (LA : OO, w/w), and shell concentration of 1 mg mL^−1^. Hybrid nanoparticles were subsequently synthesized using these optimized parameters for further characterization.

### 3.3. Synthesis and characterization of hybrid nanoparticles with selected parameters

Using the optimized parameters identified by the Taguchi analysis, the optimal HNP formulation was successfully prepared for further characterization. First, scanning transmission electron microscopy (STEM) was used to image the morphological structure of HNPs, and spherical shapes of the nanoparticles were supported (Fig. 4A). Furthermore, the optimized HNPs were characterized by DLS, confirming a particle size below 200 nm. The nanoparticles exhibited an average size of 187.7 ± 2.4 nm and a PDI of 0.06 ± 0.02 (Fig. 4B) with a solid content of 9.45 mg mL^−1^. Also, they exhibited good colloidal stability with a zeta potential value of −36.7 ± 0.4 at pH 6.3. These findings are consistent with our previous study, which reported a similar average particle size (≈217 nm), supporting the reproducibility of surfactant-free hybrid nanoparticle systems.^34^ HNPs were further characterized by pH-dependent zeta potential analysis to evaluate their surface charge behavior under different pH conditions (Fig. 4C and D). The nanoparticles exhibited positive zeta potential values below pH 4 and negative values above pH 4. The observed charge shift indicates an isoelectric point close to that of BSA (pI ≈ 4.7),^46^ consistent with BSA being the predominant component of the BSA : HA shell. Particle size and PDI increased around the isoelectric point, likely due to reduced electrostatic repulsion and subsequent particle aggregation. To better understand the surface properties of HNPs, pH-dependent zeta potential measurements were also performed for pure BSA, pure HA, and the BSA : HA conjugates (Fig. S6). HA exhibited negative zeta potential values across the entire pH range due to its polyanionic nature and did not display a distinct isoelectric point.^47^ In contrast, BSA showed a PI around pH 4.2, consistent with the literature.^46^ The BSA : HA conjugate exhibited a similar PI, indicating that BSA is the main contributor to the surface charge behavior of HNPs and supporting the formation of the conjugate shell. Our findings are consistent with those of previously reported surfactant-free hybrid nanosystems and support the use of BSA : HA conjugates as an alternative to synthetic surfactants.^11^

**Fig. 4:**
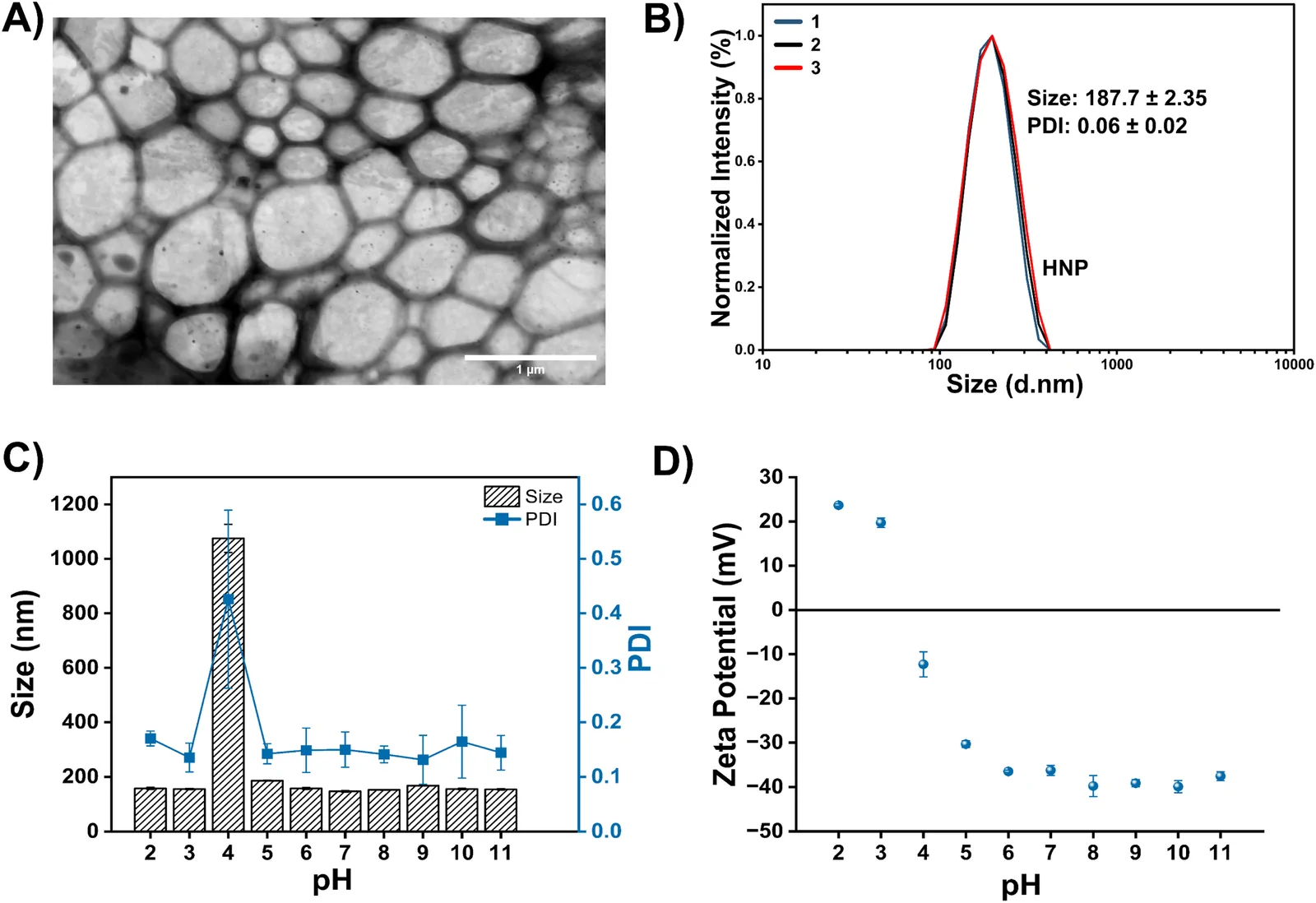
Physicochemical characterization of HNPs. (A) Scanning transmission electron microscopy (STEM) images of HNPs, (B) size normal distribution of HNPs, and (C) pH dependence of hydrodynamic diameter (nm), polydispersity index, and (D) zeta potential (mV) of HNPs.

Furthermore, TW20-stabilized nanoparticles were also synthesized as a control formulation to compare the physicochemical performance of the proposed surfactant-free HNPs with a conventional surfactant-based system. Hydrodynamic size, PDI, and zeta potential were subsequently evaluated. (Fig. S7). STEM images also confirmed their morphological structures (Fig. S8). HNPs exhibited a smaller particle size than CNP2 (327.8 ± 2.5 nm; PDI: 0.15 ± 0.01), which was formulated with 0.1% TW20 corresponding to the shell concentration used in HNPs. CNP1, prepared with a higher TW20 concentration (1%), exhibited a particle size of 161.4 ± 2.0 nm and a PDI of 0.11 ± 0.01. It was observed that the particle size decreased with increasing surfactant content, which is consistent with the literature.^48^ The CNPs exhibited a comparable pH-dependent zeta potential profile (Fig. S9). Both HNPs and CNPs remained stable over a broad pH range. Their colloidal stability likely resulted from electrostatic repulsion associated with the high absolute zeta potential values and steric stabilization provided by the BSA–HA conjugate or Tween 20. Overall, the optimized HNPs exhibited favorable physicochemical properties compared with the Tween 20-stabilized control formulations. Therefore, the optimized HNP formulation was selected for further characterization, drug encapsulation, and drug release studies.

### 3.4. Colloidal stability analysis

Understanding the stability of NLC-based hybrid platforms during storage is crucial for pharmaceutical quality control purposes. Long-term stability studies were conducted in PBS (pH 7.4) and potassium phosphate buffer at pH 6.5 and 5.8, representing physiological and tumor microenvironment pH conditions. As shown in Fig. 5, the colloidal stability of HNP and CNP formulations was monitored over 8 weeks to compare the stabilizing effect of BSA : HA conjugates with that of conventional surfactants.

**Fig. 5:**
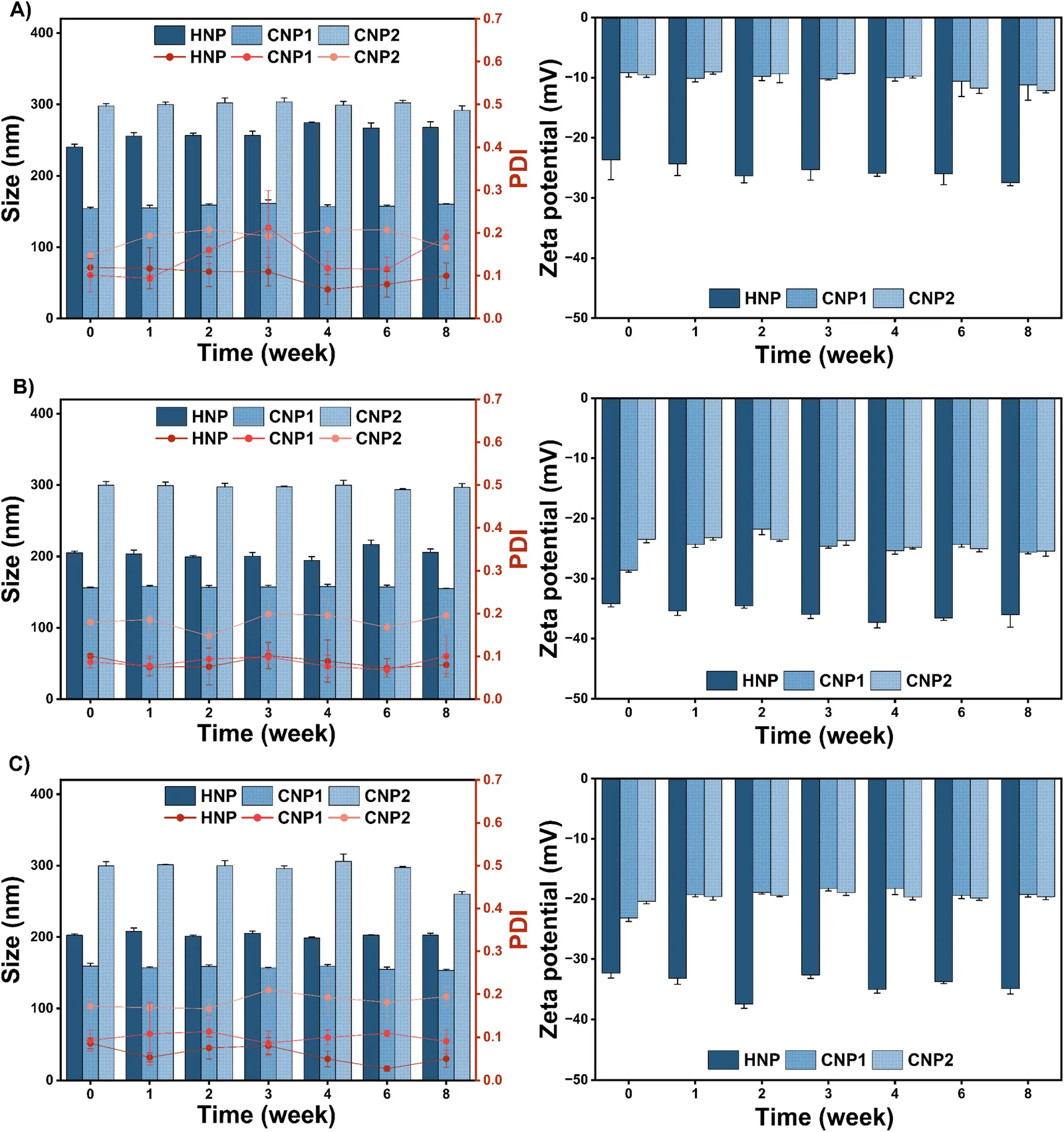
Long-term colloidal stability of nanoparticles dispersed in (A) PBS buffer (pH 7.4), (B) KPHO buffer (pH 6.5), and (C) KPHO buffer (pH 5.8) over 8 weeks, evaluated by monitoring the hydrodynamic diameter, polydispersity index (PDI), and zeta potential.

At all investigated pH values, the HNPs retained a nearly constant particle size throughout the 8-week storage period, with only minor fluctuations. The PDI values also remained low, indicating the absence of significant particle aggregation. Throughout the study, the nanoparticles retained negative surface charges, with zeta potential values of approximately ≈−20 mV in PBS and ≈−35 mV in potassium phosphate buffer. The highly negative zeta potential under both physiological and tumor-mimicking pH conditions suggests that the HNPs preserve good colloidal stability. For comparison, CNP1 and CNP2 were evaluated under the same storage conditions. Both control formulations remained stable over time under all investigated pH conditions. Although Tween 20 is a nonionic surfactant, both CNP formulations retained negative zeta potential values throughout the study. This behavior may be attributed to the preferential adsorption of hydroxyl ions at the nanoparticle–water interface.^49^ The more negative zeta potential observed in potassium phosphate buffer than in PBS is likely associated with differences in buffer ionic composition and their effects on the electrical double layer.^50^ Overall, the comparable stability of HNPs and CNPS demonstrates that the BSA : HA conjugate shell provides an effective stabilization strategy through the combined effects of steric and electrostatic interactions. These findings highlight the potential of BSA : HA conjugates as a promising surfactant-free alternative to conventional synthetic surfactants for the stabilization of lipid-based nanocarriers.

Moreover, colloidal stability was further evaluated for HNPs under physiologically relevant conditions by incubating the nanoparticle formulations at 37 °C in both PBS and complete growth medium (CGM) (Fig. S10). This experimental condition was used to mimic *in vitro* biological testing conditions. The results demonstrated that no significant changes in hydrodynamic size or PDI were observed during incubation. These findings indicate that the HNP system retained its colloidal stability under biologically relevant temperature and medium conditions, supporting its suitability for subsequent *in vitro* cellular studies.

### 3.5. Doxorubicin encapsulation and *in vitro* drug release

DOX-HCl was loaded into HNPs at a 0.5 mg per mL concentration while synthesizing the nanoparticles (DOX-HNPs). The encapsulation process was carried out through the mini-emulsion solvent evaporation method by adding the lipid phase to the DOX-HCl solution before mixing the continuous phases. After solvent evaporation and the final centrifugation, as mentioned before, they were transferred to PBS (pH 7.4) or potassium phosphate buffers (pH 6.5–5.8) to match the pH of the release medium. The encapsulation efficiency of DOX-HNPs was calculated to be 67.6 ± 4.0% using a calibration curve shown in Fig. S3 and the DOX concentration of DOX-HNPs was found to be 337.8 ± 20.1 µg mL^−1^. Moreover, drug loading content was found to be 4.47 ± 0.26% according to encapsulation efficiency. Fig. S11A demonstrates successful DOX-HCl encapsulation into HNPs by UV-vis spectroscopy, revealing a clear absorbance peak at the maximum wavelength of DOX (*λ*_max_ = 480 nm). FTIR analysis (Fig. S11B) further confirmed the successful incorporation of the drug into the nanoparticle structure.

DOX encapsulation resulted in a slight increase in hydrodynamic size (from 187.7 ± 2.35 to 216.9 ± 5.6 nm, Fig. 4 and 6), while the PDI remained low (0.13 ± 0.04, Fig. 6), indicating that the nanoparticles preserved their favorable colloidal characteristics after drug loading. Following centrifugation to remove free drug, the DOX-HNPs were redispersed in PBS (pH 7.4) or potassium phosphate buffer (pH 6.5 or 5.8), and their hydrodynamic size was measured by DLS. The particle size was 238.3 ± 4.4 nm in PBS (pH 7.4), remained largely unchanged at pH 6.5 (234.4 ± 2.2 nm), and increased moderately to 264.4 ± 0.73 nm at pH 5.8. The observed minor size variations are acceptable and do not affect the colloidal stability of the system, since PDI values stay below <0.2. This indicates that HNPs retain structural integrity under all conditions. Moreover, the consistently high absolute zeta potential values of DOX-HNPs across all tested pH conditions further supported their colloidal stability (Fig. 6B).

**Fig. 6:**
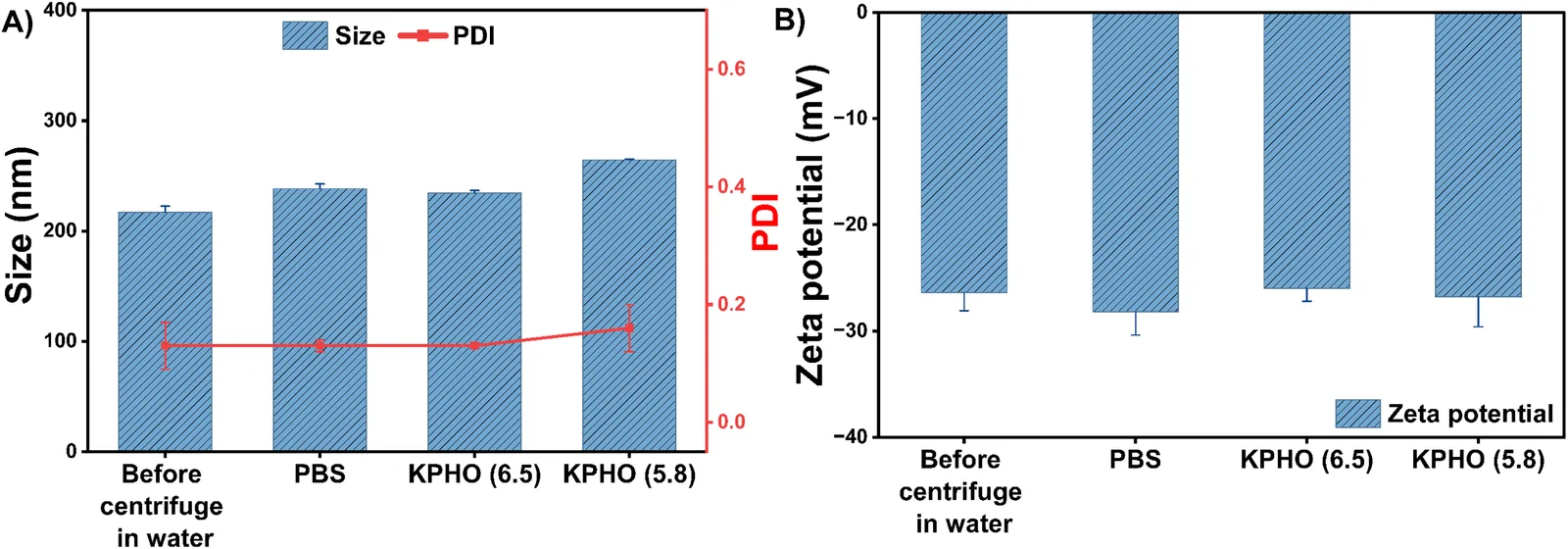
Physicochemical characterization of DOX-HNPs: (A) hydrodynamic diameter (nm), polydispersity index (PDI), and (B) zeta potential (mV) values of DOX-HNPs measured in different media, including water before centrifugation, phosphate-buffered saline (PBS, pH 7.4), and potassium phosphate (KPHO) buffers at pH 6.5 and pH 5.8 after centrifugation.

After confirming the structural integrity and stability of DOX-HNPs, drug release studies were carried out to assess their stimuli-responsive behavior under various conditions. HNPs' responsiveness to external and microenvironmental stimuli, such as temperature, pH, and enzymatic activity, was studied. PBS (pH 7.4) was used to mimic physiological pH, while potassium phosphate buffers (pH 6.5 and 5.8) were used to mimic the pH of the tumor microenvironment and endosomal compartments, respectively. Fig. 7 shows the cumulative release of free DOX and DOX release characteristics of DOX-HNPs at three pH levels (5.8, 6.5, and 7.4), at two temperatures (37–50 °C), and with/without enzyme conditions over 96 hours. As an enzyme, proteinase K (*Tritirachium album*) is used to degrade the BSA portion of the HNP shell. The release dynamics remained constant across all three groups in identical experimental settings. As expected, free DOX release reached approximately 100% in about 2 hours under all conditions (Fig. 7A and B), emphasizing the need for a controlled release system to improve the therapeutic performance of DOX-HCl.

**Fig. 7:**
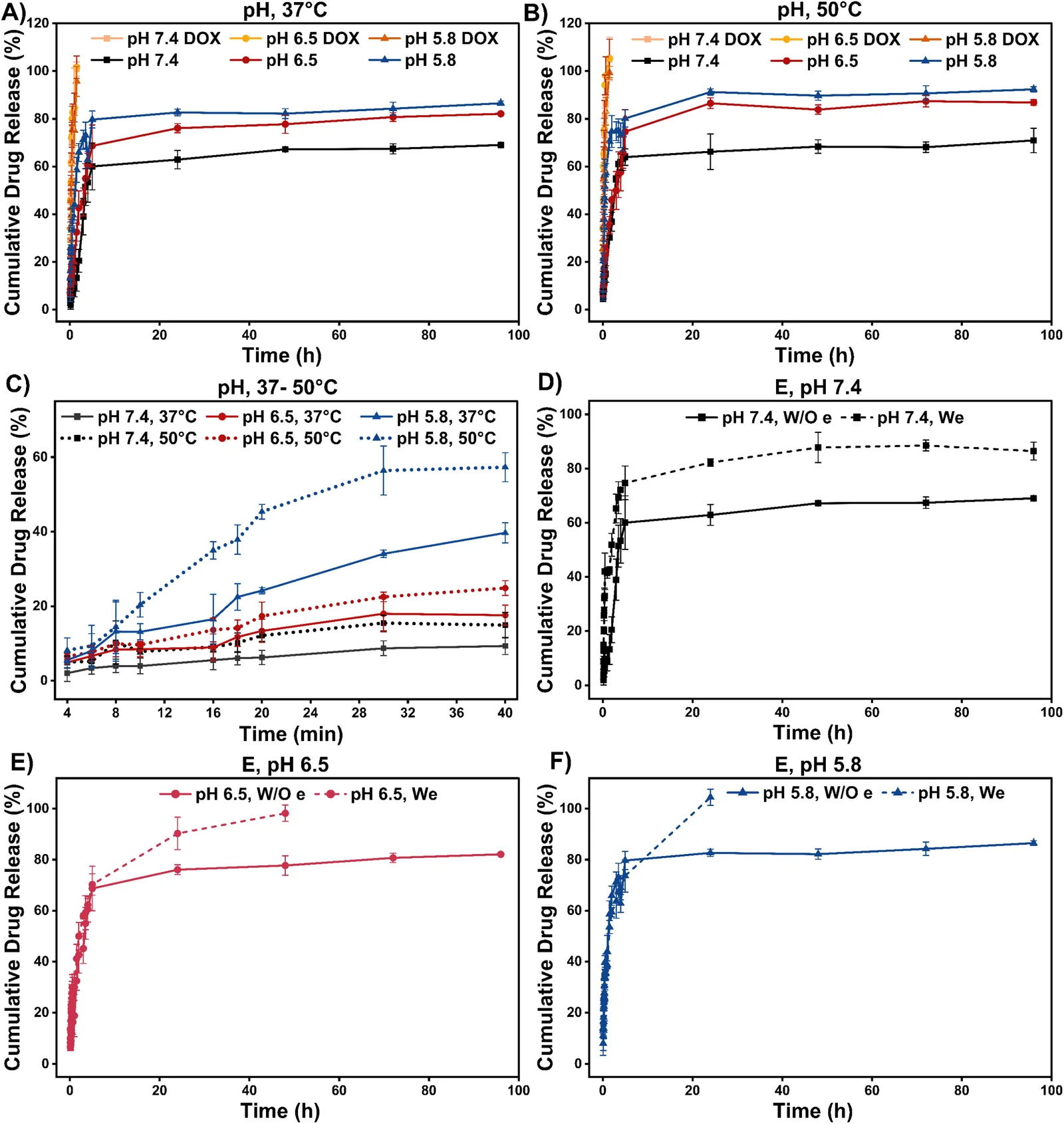
Cumulative release profiles of DOX from DOX-HNPs under different pH, temperature, and enzymatic conditions, demonstrating stimulus-responsive release behavior. (A) Comparative cumulative release profiles of free DOX and DOX released from DOX-HNPs at 37 °C and at pH 7.4, pH 6.5 and pH 5.8, (B) comparative cumulative release profiles at 50 °C at pH 7.4, pH 6.5 and pH 5.8, (C) comparative short-term cumulative release profiles of DOX-HNPs at 37 °C and 50 °C under all tested pH conditions, (D) DOX release at pH 7.4 in the absence and presence of proteinase K, (E) DOX release at pH 6.5 in the absence and presence of proteinase K, and (F) DOX release at pH 5.8 in the absence and presence of proteinase K. Data are presented as mean ± standard deviation (SD) (*n* = 3).

The release experiments were designed to evaluate the contribution of the BSA : HA conjugate to pH-responsive drug release and the effect of heating the lipid core above its melting point (50 °C) on potential temperature-triggered drug release. Fig. 7A depicts drug release profiles at various pH levels at physiological temperature (37 °C), whereas Fig. 7B depicts release behavior at increased temperatures (50 °C). The total release percentages at pH levels 7.4, 6.5, and 5.8 were found to be 69.0 ± 0.4%, 82.1 ± 1.6%, and 86.5 ± 0.9%, respectively, after 96 h at 37 °C. The relatively increased release under acidic conditions demonstrates the pH-sensitivity of the HNP system. At 50 °C, a similar pattern was observed, with cumulative drug release at pH 7.4, 6.5, and 5.8 reaching 71.0 ± 5.1%, 86.8 ± 1.3%, and 92.3 ± 1.1%, respectively. Consistent with our previous findings on HA nanocapsules, the current formulation exhibited a similar pH-dependent drug release behavior.^19^ These results may be attributed to the structural rearrangement of BSA together with increased hydration and chain mobility of HA, which may increase nanoparticle shell permeability and facilitate drug diffusion. This behavior is consistent with the well-established role of HA-functionalized nanocarriers in triggering pH-responsive release.^51,52^

Over the 96-hour release period, temperature had only a limited effect on drug release under all investigated pH conditions, although a slightly greater effect was observed under acidic conditions. Therefore, short-term release studies were additionally performed to better evaluate the influence of temperature on drug release, as commonly reported in the literature.^53^ In Fig. 7C, the initial release at all pH levels within the first 40 minutes is presented. At all pH values, cumulative drug release was higher at 50 °C than at 37 °C (Fig. 7C). The largest temperature-induced increase was observed at pH 5.8, where cumulative drug release increased from 39.6 ± 2.7% to 57.2 ± 3.9%, indicating that the temperature-triggered release behavior was more pronounced under acidic conditions. The observed increased release within the initial 40 min reflects a rapid early-phase response of the nanocarrier system to external temperature, such as localized hyperthermia in cancer therapy. Since hyperthermia is typically achieved within a short time window following treatment, the ability of the system to respond at early points may contribute to improved therapeutic efficiency by enabling prompt activation of drug release at the target site. Such behavior is advantageous for cancer treatment strategies relying on externally triggered and temporally controlled drug delivery. Importantly, the enhanced release observed at 50 °C can be attributed to the thermosensitive nature of the LA on the lipid core. At temperatures exceeding the lipid melting point, increased lipid fluidity facilitates structural rearrangement and enhances drug diffusion.^24^ This behavior is relevant to hyperthermia-triggered drug delivery, where localized heating is used as an external stimulus to enhance drug release.^32^ The higher drug release observed at 50 °C is consistent with increased lipid fluidity above the lipid phase transition temperature.

In the cancer microenvironment, proteases are frequently overexpressed and promote tumor invasion and metastasis.^31^ Therefore, enzyme-responsive drug delivery systems may offer a potential approach for exploiting proteolytic activity to facilitate drug release under protease-rich conditions. Proteinase K (*Tritirachium album*) is widely used in biomaterials science as a model protease for protein degradation. In this study, proteinase K enzyme of 8 U mg^−1^ (with a protein/enzyme ratio of 2 : 1 (w/w)) was used to digest the protein portion of the biopolymer shell of DOX-HNPs to increase drug release.

An enzyme-responsive release study was performed at 37 °C for 96 hours. After 96 hours, the cumulative release of DOX-HNPs at pH 7.4 reached 69.0 ± 0.4% in the absence of enzyme and 86.4 ± 3.3% in its presence (Fig. 7D). At pH 6.5, proteinase K further increased the cumulative drug release from 82.1 ± 1.6% to 98.2 ± 3.2% after 48 h (Fig. 7E). Similarly, at pH 5.8, cumulative drug release reached 86.5 ± 0.9% after 96 h without proteinase K, whereas 104.4 ± 5.4% was achieved in the presence of the enzyme after only 24 h (Fig. 7F). Enzymatic triggering resulted in an approximately 18% increase in drug release compared with enzyme-free conditions across all pH values, indicating a consistent enzyme-responsive release behavior. These findings are consistent with our previous research where a serine protease-triggered system showed roughly 15% and 10% increases in drug release at pH 6.5 and 7.4, respectively.^34^ These findings indicate that the proteinase K enzyme broke down some portion of the BSA into smaller amino acids, allowing DOX to be released more easily into the environment. Furthermore, the rapid release observed under decreasing pH conditions confirms the system's dual responsiveness, indicating sensitivity to both enzymatic degradation and acidic microenvironmental conditions. Therefore, the DOX-HNP system shows promise as a controlled-release platform for DOX delivery under different conditions.

Understanding and enhancing therapeutic efficacy requires kinetic modeling of the drug release profile. We analyzed drug release data at all pH levels (5.8, 6.5, and 7.4) at both temperatures (37–50 °C) using kinetic models such as zero-order, first-order, Higuchi, and Korsmeyer–Peppas to understand the drug release mechanism of the DOX-HNPs better. The kinetic models were used for the first 60% of drug release, as the Korsmeyer–Peppas model is only theoretically valid within this range.^54^ Fig. S12 depicts the model plots for both temperatures and all pH values, while kinetic modeling analyses performed for the enzyme-responsive release system at different pH values are presented in Fig. S13. Table S5, on the other hand, contains the correlation coefficients (*R*^2^) for all conditions obtained from the linearization of the model data.

The drug release kinetics of DOX-HNPs varied significantly depending on pH, temperature, and enzyme presence, demonstrating systematic behavior when fitted to several mathematical models. The selected kinetic models effectively explain the release behavior, as evidenced by the high *R*^2^ values observed under all conditions. At pH 7.4 and without enzymes, the system exhibited a strong match to the zero-order (*R*^2^ = 0.97–0.98) and first-order (*R*^2^ = 0.96–0.97) models, which remained stable at 37 °C and 50 °C. This indicates that under physiological pH conditions, the system exhibits a relatively steady and controlled release profile, with minimal fluctuations over time. The Higuchi model retained a high level of agreement (*R*^2^ = 0.91–0.94), confirming that diffusion contributes significantly to the release kinetics alongside other mechanisms. Increasing the temperature to 50 °C at pH 7.4 enhances diffusion and polymer relaxation in the Korsmeyer–Peppas model, resulting in an *R*^2^ value of 0.94 to 0.96. In contrast, at pH 6.5 and 5.8, the system showed a clear change in behavior, where the Higuchi and Korsmeyer–Peppas models became more suitable to describe the release profile. At pH 6.5 in the absence of enzymes, both the Higuchi and Korsmeyer–Peppas models provided excellent fits across 37 °C and 50 °C, with *R*^2^ values of 0.97 and 0.96–0.98, respectively. This indicates a clear shift toward a diffusion-controlled matrix release mechanism.^54^ When the pH decreased to 5.8, the Higuchi and Peppas models remained dominant (*R*^2^ = 0.90–0.91), indicating that diffusion remained the primary control mechanism.

When the influence of enzyme presence was investigated, a significant difference was found, particularly at pH 7.4. At 37 °C in the presence of enzymes, both zero-order (*R*^2^ = 0.66) and first-order (*R*^2^ = 0.73) models showed poor fitting, suggesting a loss of regular release kinetics. The Higuchi (*R*^2^ = 0.77) and Korsmeyer–Peppas (*R*^2^ = 0.78) models provided a better fit, indicating a mixed process of diffusion and degradation for release. At pH 6.5 and 5.8, enzymes did not eliminate model fits, but they weakened the performance of zero-order and first-order models, while the Higuchi and Korsmeyer–Peppas models still described the system more effectively.

The *n* values obtained from the Korsmeyer–Peppas model generally ranged between 0.48 and 0.75. This indicates that the release mechanism cannot be explained solely by Fickian diffusion but also involves processes such as polymer swelling and chain relaxation.^55^ In enzymatic release, the decrease in *n* values to 0.48 at pH 7.4 indicates that diffusion plays a role alongside enzyme-driven matrix degradation during release. The *n* values at pH 6.5 and 5.8 (≈0.48–0.53) indicate anomalous release, with minimal contribution from enzymatic degradation due to lower enzyme activity outside its optimal pH range.^56^ At pH 7.4, however, the poorer fit in the enzymatic models indicates a greater contribution of enzymatic degradation. Overall, the developed DOX-HNPs exhibited pH- and enzyme-triggered drug release, whereas temperature mainly enhanced the initial release phase. Collectively, these findings demonstrate that the HNPs function as a controllable multi-stimuli-responsive drug delivery platform.

Furthermore, to evaluate the effect of release conditions on nanoparticle stability, DOX-HNPs' hydrodynamic sizes were remeasured after release experiments conducted in enzyme-free media. As shown in Fig. S14, only limited changes in nanoparticle sizes were observed under different pH and temperature conditions. The average particle size, initially 238.3 ± 4.4 nm at pH 7.4, was determined to be 254.1 ± 2.1 nm and 256.0 ± 2.6 nm at 37 °C and 50 °C, respectively. Similarly, at pH 6.5, the initial size was 234.4 ± 2.2 nm, while, after releasing, it was measured to be 235.6 ± 1.7 nm at 37 °C and 245.7 ± 7.0 nm at 50 °C. At pH 5.8, the initial particle size of 264.4 ± 0.7 nm decreased to 244.1 ± 7.7 nm after 37 °C and was determined to be 258.4 ± 1.5 nm at 50 °C. Overall, the relatively minor changes in particle size indicate that the nanoparticles largely preserved their structural integrity throughout the release experiments despite exposure to different pH values and temperatures. These findings are consistent with a predominantly diffusion-driven release mechanism, with the differences in release profiles likely arising from changes in diffusion pathways and polymer–medium interactions under the tested conditions.

### 3.6. Cell cytotoxicity studies

After evaluating the drug release behavior of HNPs, cell viability assays were performed as a follow-up study. The effects of HNPs and DOX-HNPs on cell viability were assessed in MCF-7 breast cancer cells and L929 mouse fibroblast cells using the MTT assay after 24 and 48 hours of incubation. For L929 cells, the assay was conducted according to the ISO 10993-5:2020 standard.

The results demonstrated that HNP formulations retained relatively high cell viability within the examined concentration range (10–800 µg mL^−1^). For DOX-HNPs, the corresponding DOX concentration range was 0.447–35.760 µg mL^−1^, calculated based on the experimentally determined drug loading of 4.47%. As presented in Fig. 8, L929 cell viability remained between ≈80 and 111% after 24 h and ≈70–96% after 48 h of incubation. The preservation of approximately 70.5% viability even at prolonged exposure indicates that HNPs did not induce significant cytotoxicity in fibroblast cells and remained within acceptable biocompatibility limits. Although a partial reduction in viability was observed at higher concentrations and longer incubation periods, this effect appeared to be concentration- and time-dependent.

**Fig. 8:**
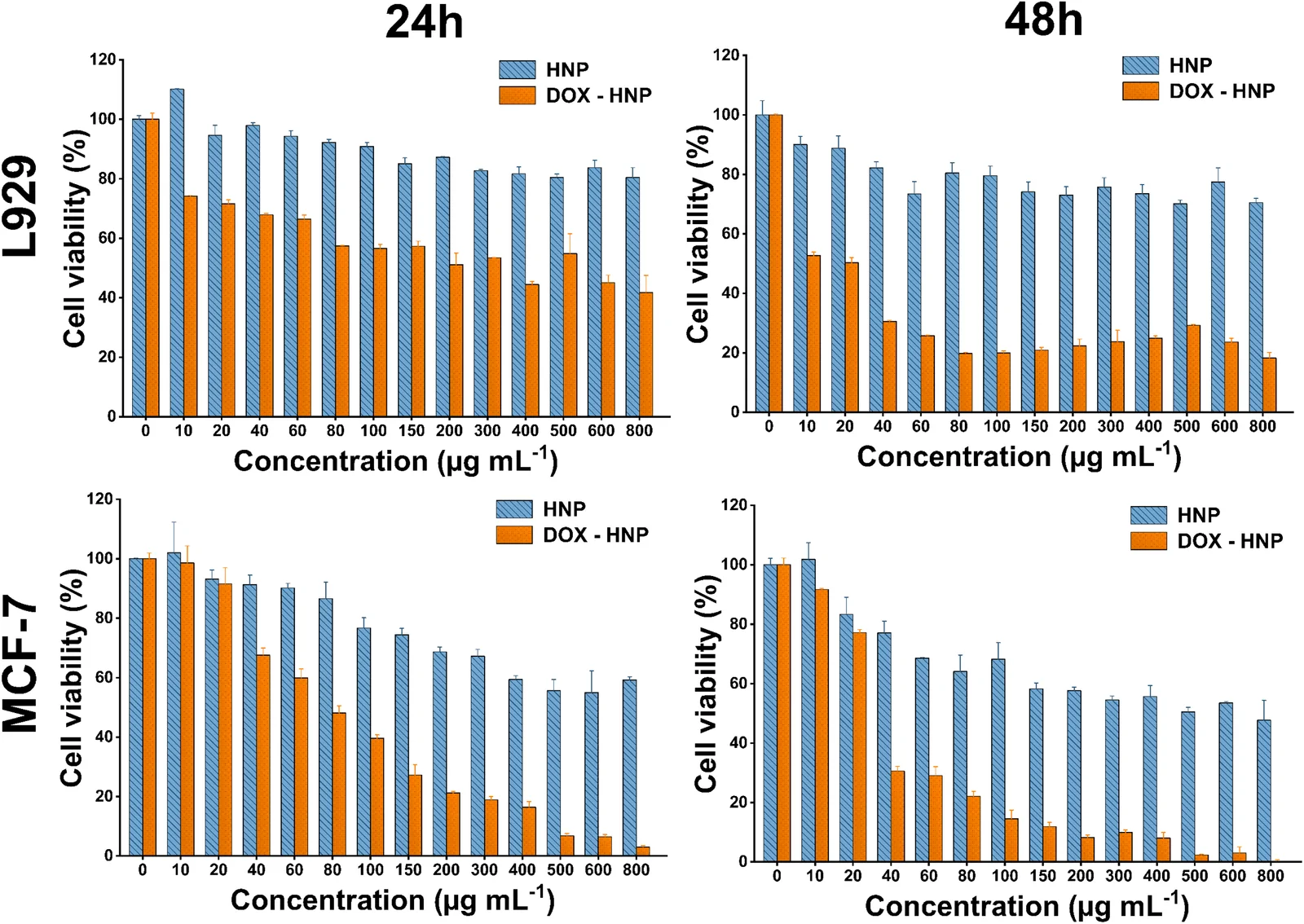
Cell viability of L929 mouse fibroblast cells and MCF-7 breast cancer cells following treatment with HNPs and DOX-HNPs for the indicated incubation period, as determined by the MTT assay. The results demonstrate the comparative cytotoxic effects of blank HNPs and DOX-HNPs on normal and cancer cell lines. Data are expressed as mean ± standard deviation (SD) (*n* = 3).

In contrast, HNP treatment resulted in lower viability values in MCF-7 cells compared with L929 fibroblasts. At the maximum concentration, viability decreased to 59 ± 1.1% after 24 h and further declined to 48 ± 6.7% after 48 h. This difference may be associated with the inherent affinity of HA present on the nanoparticle surface toward cancer cells, as well as the relatively higher endocytic activity of MCF-7 breast cancer cells. In addition, HA-based nanosystems have been reported to interact more effectively with CD44-expressing breast cancer cells, which may enhance cellular uptake and consequently contribute to increased intracellular stress and reduced cell viability. Similar observations have previously been reported for HA-coated nanoparticle systems in breast cancer models.^57^

DOX-loaded formulations exhibited considerably stronger cytotoxic effects in both cell lines. Following DOX-HNP treatment, L929 cell viability ranged between ≈81 and 41% after 24 h and ≈62–18% after 48 h, depending on concentration. However, MCF-7 cells showed substantially greater sensitivity under the same experimental conditions, with viability decreasing to ≈2% after 24 h and ≈0.2% after 48 h. The enhanced cytotoxic response in MCF-7 cells may be associated with nanoparticle-mediated intracellular delivery of DOX, increased drug release under endosomal pH conditions, and higher nanoparticle internalization resulting from HA-mediated cellular interactions. Comparable cell viability trends were also observed in our previous study involving HA-based nanocapsules, supporting the potential role of HA-associated interactions in the selective response of MCF-7 cells.^19^ Therefore, cell viability was observed to be relatively higher in L929.

To better understand the anticancer activity of the DOX-HNP system, the IC_50_ (half-maximal inhibitory concentration) was calculated by fitting a four-parameter log-logistic (4PL) model^58^ to the relationship between experimental cell viability and DOX-HNP concentration in both cell lines and at all incubation times, as shown in Fig. S15. DOX-HNPs demonstrated anticancer activity against MCF-7 breast cancer cells, achieving IC_50_ values of 67.4 µg mL^−1^ after 24 hours and 38.9 µg mL^−1^ after 48 hours. Previous studies have reported time-dependent cytotoxic effects of DOX-loaded HNP formulations against MCF-7 cells.^59^ These values corresponded to approximately 3.01 and 1.74 µg per mL DOX-equivalent concentrations, respectively. For comparison, a 48 h IC_50_ value of 2.43 µM free DOX (approximately 1.32 µg mL^−1^) has been reported in MCF-7 cells.^60^ The corresponding DOX-equivalent IC_50_ value of DOX-HNPs in the present study was 1.74 µg mL^−1^. Additionally, the cytotoxic activity of DOX-HNPs against L929 cells was characterized by IC_50_ values of 107.4 and 28.0 µg per mL DOX-HNPs after 24 and 48 hours, respectively. These values corresponded to approximately 4.80 and 1.25 µg per mL DOX-equivalent concentrations, respectively. Furthermore, in MCF-7 cells, cell viability decreased below 40% from 150 µg per mL DOX-HNPs onward at 24 hours and from 40 µg mL^−1^ onward at 48 hours, corresponding to approximately 6.71 and 1.79 µg per mL DOX-equivalent concentrations, respectively. Overall, the time-dependent decrease in IC_50_ values indicates increased cytotoxic activity of the DOX-HNP formulation with prolonged exposure in both cell lines.

In addition, surfactant-containing control nanoparticles (CNP1: 1% TW20 and CNP2: 0.1% TW20) were used to compare cell viability in L929 fibroblast cells (Fig. 9). These formulations were prepared to clarify the influence of surfactant concentration on cytocompatibility, particularly in relation to surfactant-free HNP systems. Due to differences in solid content between CNP1 (5.5 mg mL^−1^) and CNP2 (4.6 mg mL^−1^), the concentration range was standardized to 5–400 µg mL^−1^ to ensure consistent comparison across all formulations. In L929 cells, CNP1 showed cell viabilities of ≈−29–78% after 24 h and ≈25–74% after 48 h, whereas CNP2 exhibited ≈41–106% and ≈35–79% viability at 24 h and 48 h, respectively. In both cases, cell viability was lower than that observed for HNPs under identical experimental conditions. This finding is consistent with previous reports emphasizing the critical role of surfactant composition and concentration in determining lipid nanoparticle properties, particularly cytotoxicity and physical stability.^12^ In addition, the statistical differences between all three groups are given as a heatmap in Fig. S16. A statistically significant difference was found between HNPs and CNPs at varying concentrations. These findings demonstrate that the surfactant-free synthesis of HNPs resulted in improved cytocompatibility compared with conventionally surfactant-stabilized nanoparticles. Notably, the BSA : HA conjugate served as both a biorelevant stabilizer and a shell-forming material, enabling the fabrication of multifunctional hybrid nanoparticles.

**Fig. 9:**
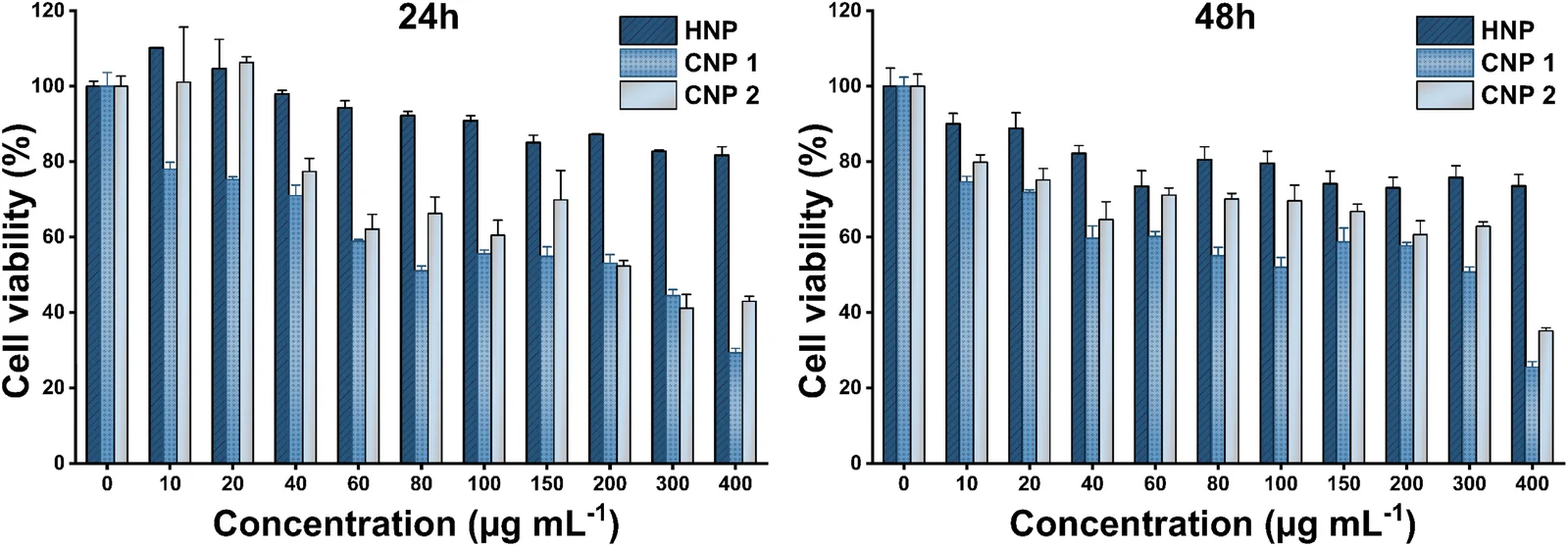
*In vitro* cytotoxicity evaluation by MTT assay. Cell viability of L929 mouse fibroblast cells following treatment with HNPs, CNP1, and CNP2, for the indicated incubation period, as determined by the MTT assay. The results demonstrate the comparative cytotoxic effects of blank HNPs and CNPs on healthy cell lines. Data are expressed as mean ± standard deviation (SD) (*n* = 3).

## 4. Conclusion

In this study, we present the successful development of surfactant-free, biopolymer-based HNPs prepared by the miniemulsion solvent evaporation method for controlled, multi-responsive DOX delivery. The shell of the nanocarrier was first constructed *via* the Maillard reaction between BSA and HA at varying weight ratios and incubation times. Thereafter, the weight ratio, incubation time, and shell concentration of the prepared conjugates with the core lipid ratio were selected as key formulation variables. Therefore, a four-level, four-factor Taguchi orthogonal design was applied to systematically investigate the hydrodynamic size of HNPs. It was observed that the increase in the weight ratio of the conjugates was identified as the most influential factor promoting the formation of smaller HNPs among the investigated formulation parameters. Thus, optimum parameters were selected as conjugate weight ratio: 6 : 1 (BSA : HA, w/w), conjugate incubation time: 96 h, lipid ratio: 1.5 (LA : OO w/w), and shell concentration: 1 mg mL^−1^. The optimized HNPs exhibited a particle size of 187.7 ± 2.4 nm, a PDI of 0.06 ± 0.02, and a zeta potential of −36.7 ± 0.4 mV, indicating a relatively narrow size distribution and favorable colloidal stability. STEM images confirmed the spherical morphology of the HNPs. Moreover, the HNPs had lower particle sizes than the corresponding surfactant-containing nanoparticles (CNP2), and both CNPs and the HNPs remained colloidally stable over an 8-week monitoring period. These findings demonstrate that the BSA : HA conjugate serves as a safe and functional stabilizer for the synthesis of lipid-based nanoparticles.

In the following stage, DOX encapsulation was achieved with an encapsulation efficiency of 67.6 ± 4.0% and a drug loading of 4.47 ± 0.26%. DOX-HNPs exhibited significantly increased drug release under acidic conditions compared with physiological pH. Under enzymatic conditions, the drug release rate increased as the pH decreased. The temperature-dependent effect on DOX release was observed within the first 40 min, suggesting a short-term effect of temperature on the release behavior. Drug release kinetics varied with the release conditions, whereas lower *R*^2^ values under enzymatic conditions indicate a more complex release mechanism due to proteinase K-induced shell degradation. Hydrodynamic size analysis after release (enzyme-free conditions) showed no significant changes, confirming that the nanoparticles remained structurally stable throughout the release. *In vitro* cytotoxicity studies demonstrated concentration- and time-dependent cytotoxicity of DOX-HNPs in MCF-7 breast cancer cells, together with a time-dependent cytotoxic response in L929 mouse fibroblasts.

Overall, the developed HNP system exhibits structural stability, enhanced cytocompatibility compared with conventionally surfactant-stabilized counterparts, and controlled DOX release under different stimuli. These findings highlight its potential as a surfactant-free, multi-responsive drug delivery platform for controlled drug release.

## Conflicts of interest

There are no conflicts to be declared.

## Supplementary Material

NA-OLF-D6NA00677A-s001

## Data Availability

The data supporting this article have been included as part of the supplementary information (SI). Supplementary information is available. See DOI: https://doi.org/10.1039/d6na00677a.
