# Supplementary material for: Multi-responsive biopolymer–lipid hybrid nanocarriers for controlled doxorubicin delivery
Source: Nanoscale Adv. 2026 Oct 9. Online ahead of print. doi: 10.1039/d6na00677a (PMC13701145; doi:10.1039/d6na00677a)
Supplement: Supplementary file 1 [file NA-OLF-D6NA00677A-s001.pdf]

Supplementary Information (SI) for Nanoscale Advances.
This journal is © The Royal Society of Chemistry

Supporting Information

Multi-Responsive Biopolymer-Lipid Hybrid Nanocarriers for Controlled Doxorubicin Delivery

Öznur Safgöl[1], Banu Iyisan[1,2*]

O. Safgol, B. Iyisan

[1]Biofunctional Nanomaterials Design (BIND) Laboratory, Institute of Biomedical Engineering, Bogazici University, 34684, Istanbul, Turkey

[2]Center for Targeted Therapy Technologies (CT3), Bogazici University, 34684, Istanbul, Turkey

*E-mail: banu.iyisan@bogazici.edu.tr

Table S1. Composition and Protein Content of Synthesized Maillard Reaction Conjugates

Weight ratio of BSA: HA (w/w)	Incubation time of conjugates (h)	Maillard Conjugate	Protein Concentration (µg/mL)
1:1	24	1.1.24	393.0±88.0
	48	1.1.48	460.2±83.8
	72	1.1.72	448.3±95.6
	96	1.1.96	482.0±81.0
3:1	24	3.1.24	640.0±94.3
	48	3.1.48	619.8±91.5
	72	3.1.72	681.0±115.5
	96	3.1.96	625.5±93.7
5:1	24	5.1.24	669.8±99.2
	48	5.1.48	725.5±97.4
	72	5.1.72	762.7±106.2
	96	5.1.96	814.2±94.1
6:1	24	6.1.24	737.0±84.3
	48	6.1.48	725.5±97.4
	72	6.1.72	777.3±91.0
	96	6.1.96	739.7±92.4

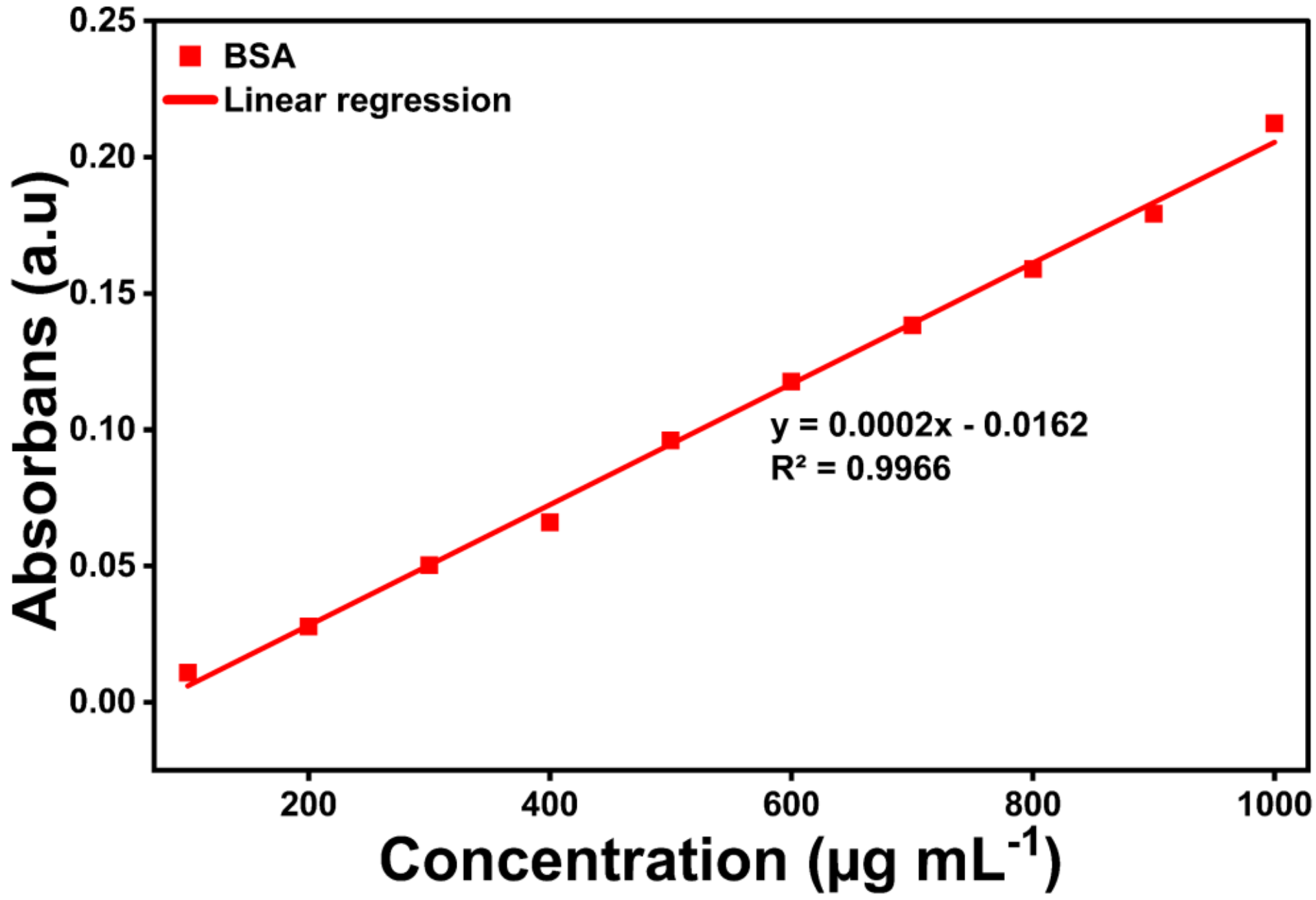

Figure S1. Calibration Curve of BSA to calculate protein concentrations (Protein Assay) of BSA: HA conjugate

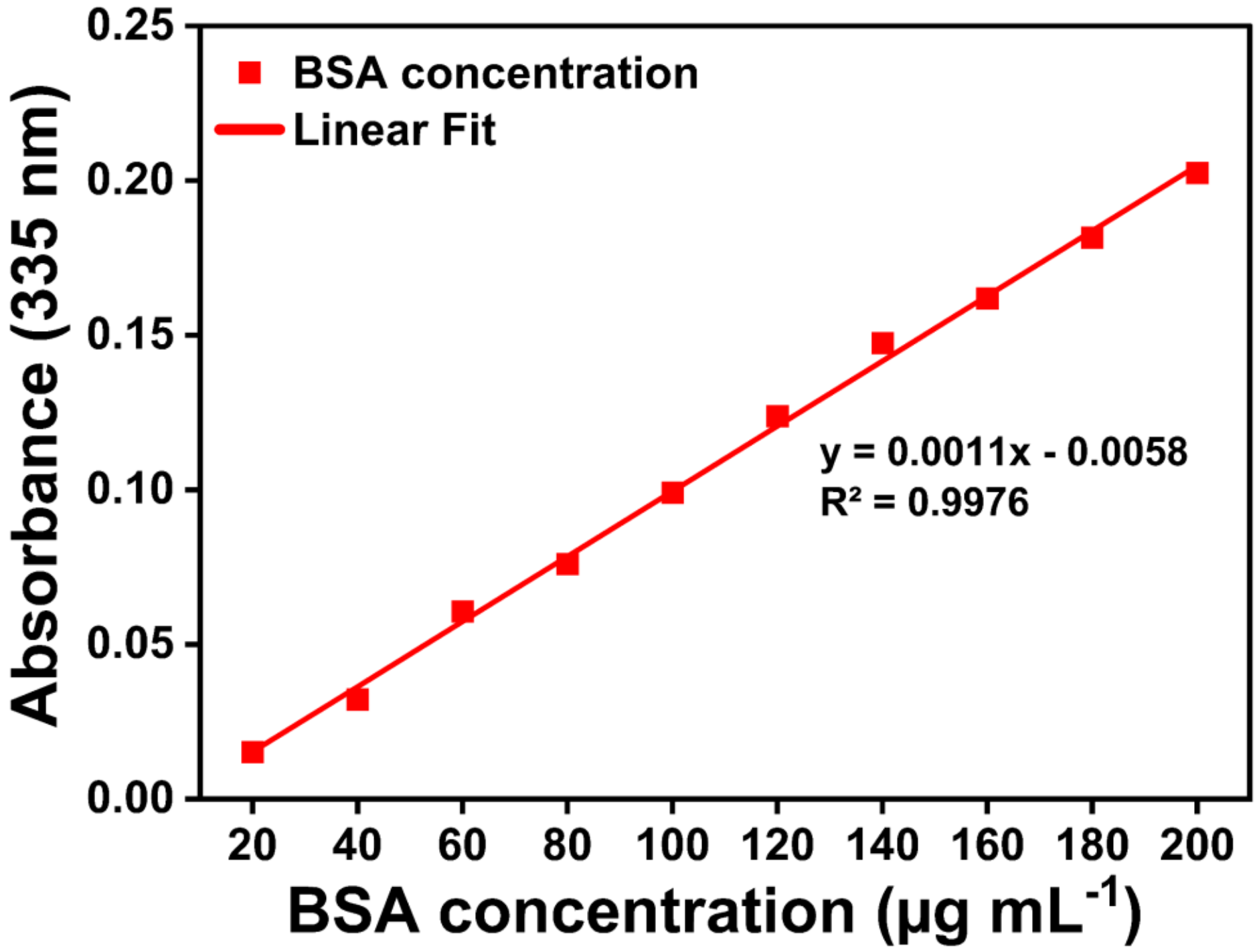

Figure S2. Calibration curve for the determination of primary amino group concentration using the TNBS assay

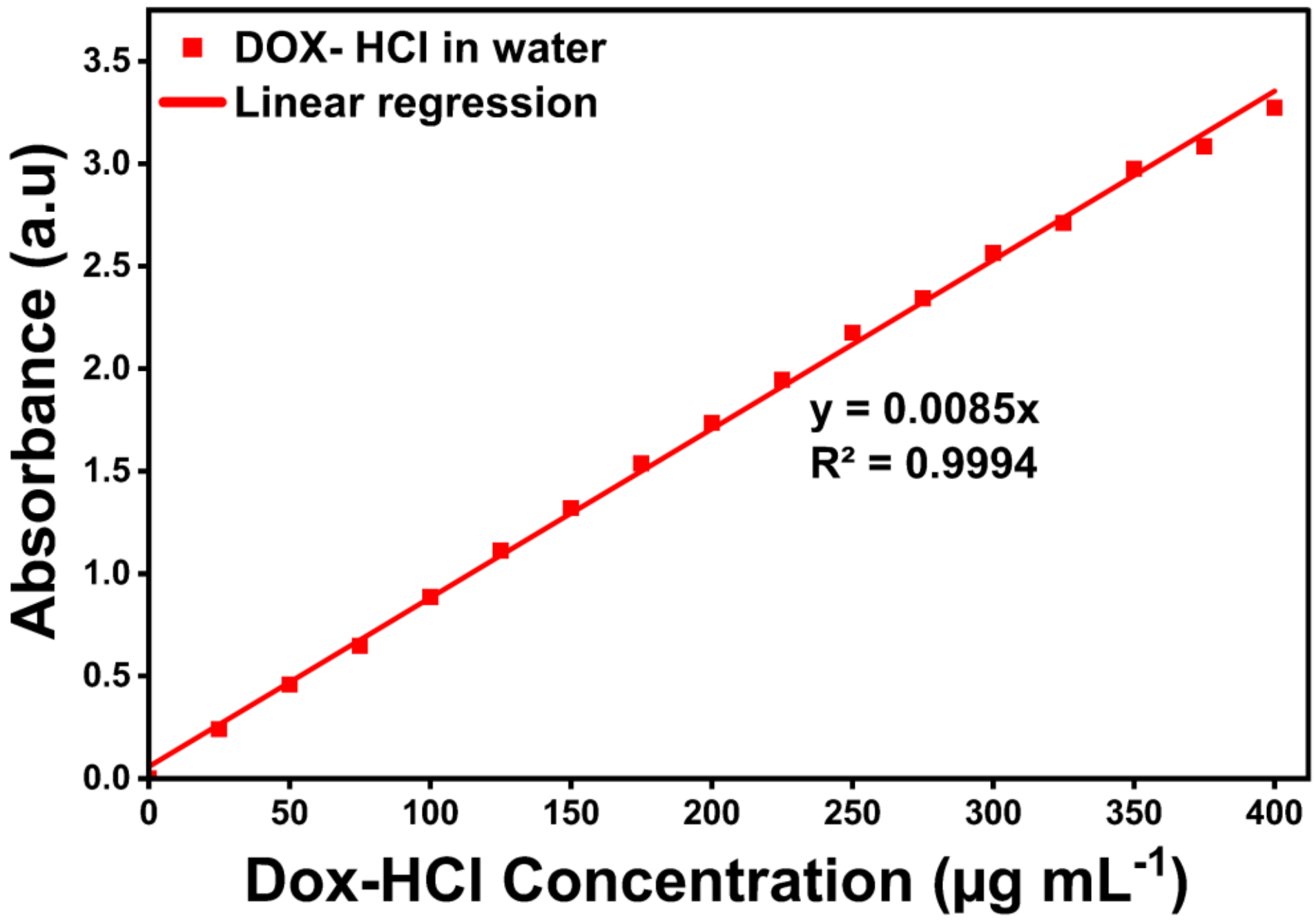

Figure S3. Calibration curve of DOX-HCl in water for calculating encapsulation efficiency

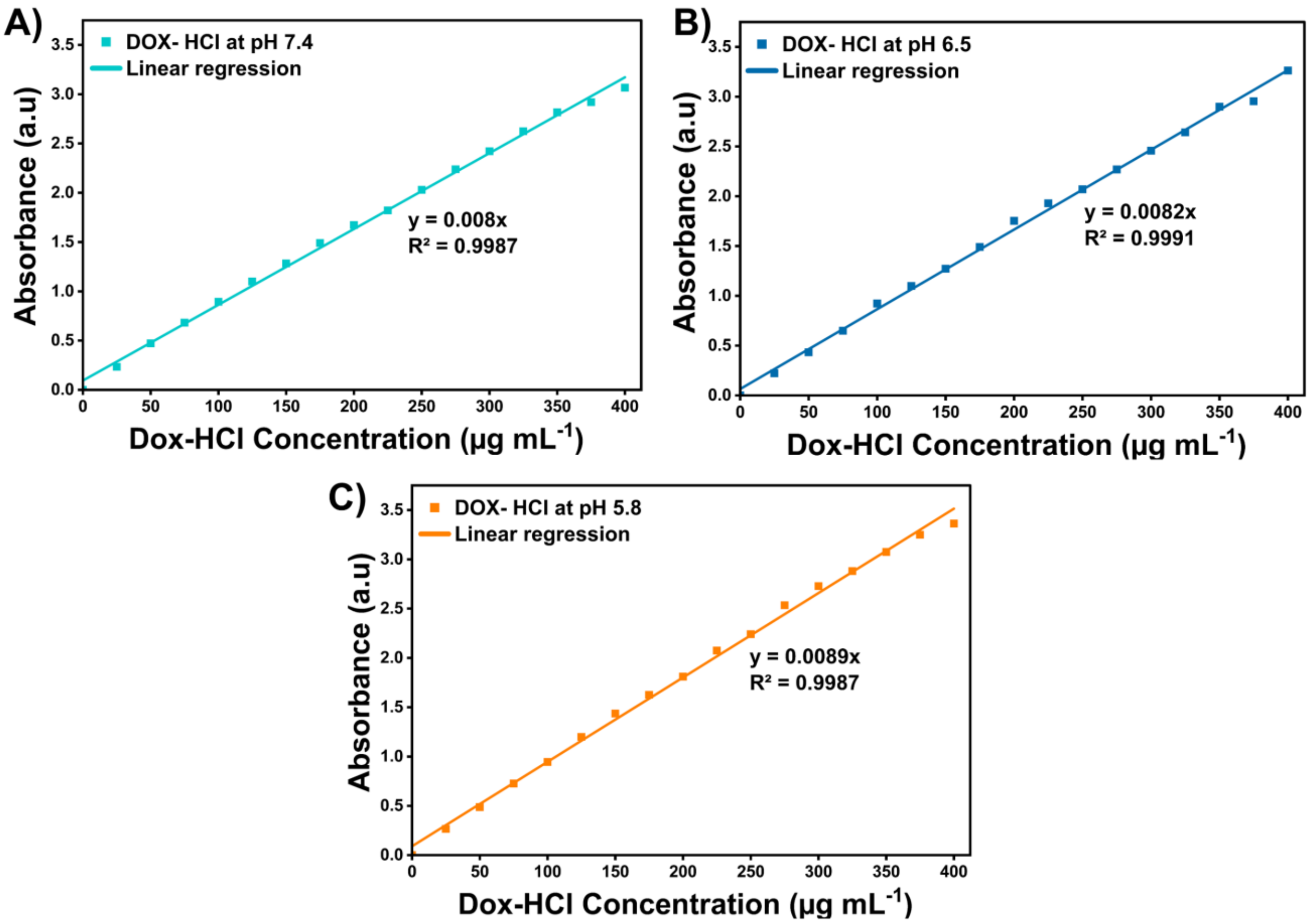

Figure S4. Calibration curves of DOX-HCl in different buffers: A) PBS (pH 7.4), B) KPHO (pH 6.5), C) KPHO (pH 5.8)

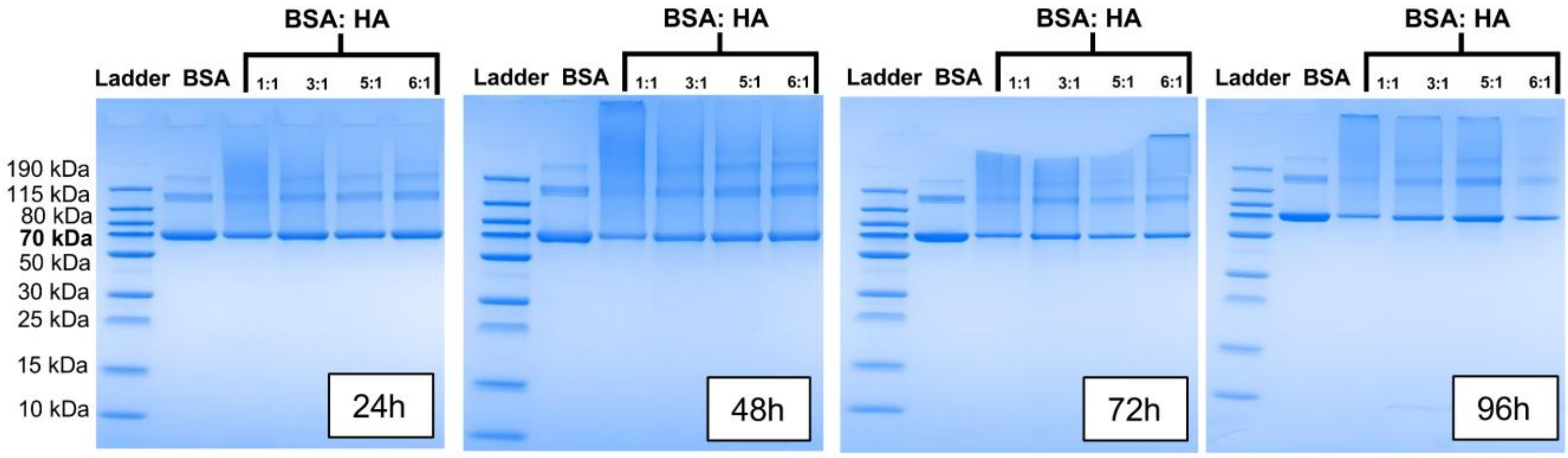

Figure S5. SDS-PAGE characterization results of BSA-HA complexes with different weight ratios and incubation time

TableS2. Degree of conjugation of BSA: HA conjugates at different weight ratios and incubation times

Weight ratio (BSA: HA, w/w)	Degree of conjugation (%)			
	Incubation time (h)			
	24	48	72	96
1:1	21.4±1.7	33.7±5.5	33.7±5.8	33.5±7.7
3:1	30.0±0.7	33.8±7.2	37.6±7.3	44.9±1.8
5:1	34.3±1.3	37.2±6.7	41.3±1.3	44.3±0.8
6:1	43.3±3.3	45.7±2.7	50.9±3.0	60.3±2.1

Table S3. Response table for means

**Level**	**Conjugate weight ratio (BSA: HA, (w/w))**	**Incubation time (h)**	**Lipid weight ratio (LA: OO, w/w))**	**Shell concentration (mg mL^{-1})**
1	301.6	274.2	278.4	269.2
2	262.3	271.0	282.3	254.1
3	246.6	270.0	241.2	263.8
4	238.6	233.6	233.6	261.9
Delta	63.0	40.6	40.6	15.1
**Rank**	1	3	2	4

Delta: difference between the maximum and minimum level means. Rank: relative importance of each factor based on Delta

Table S4. Analysis of variance for hydrodynamic size

Source	DF	Seq SS	Adj SS	Adj MS	F-Value	p-Value	η²
Conjugate weight ratio (w/w)	3	9412.8	9412.8	3137.6	4.75	0.117	0.435
Incubation time (h)	3	4404.9	4404.9	1468.3	2.22	0.265	0.204
Lipid weight ratio (LA: OO w/w)	3	5350.5	5350.5	1783.5	2.70	0.218	0.248
Shell concentration (mg mL^{-1})	3	467.2	467.2	155.7	0.24	0.867	0.022
Error	3	1983.3	1983.3	661.1			0.092
Total	15	21618.6	21618.6				

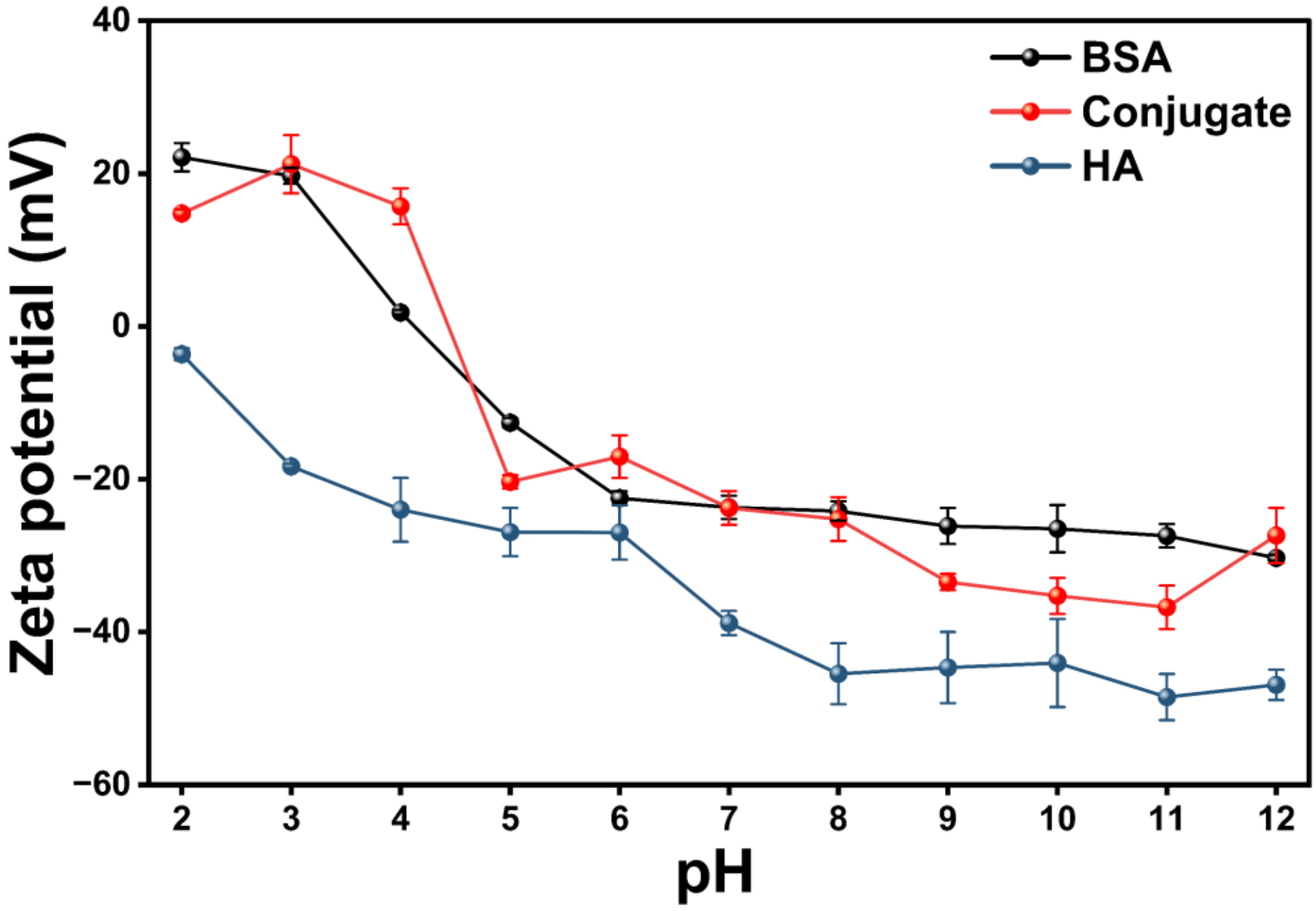

Figure S6. pH-dependent zeta potential of pure BSA, HA, and BSA: HA (6:1, w/w) conjugate

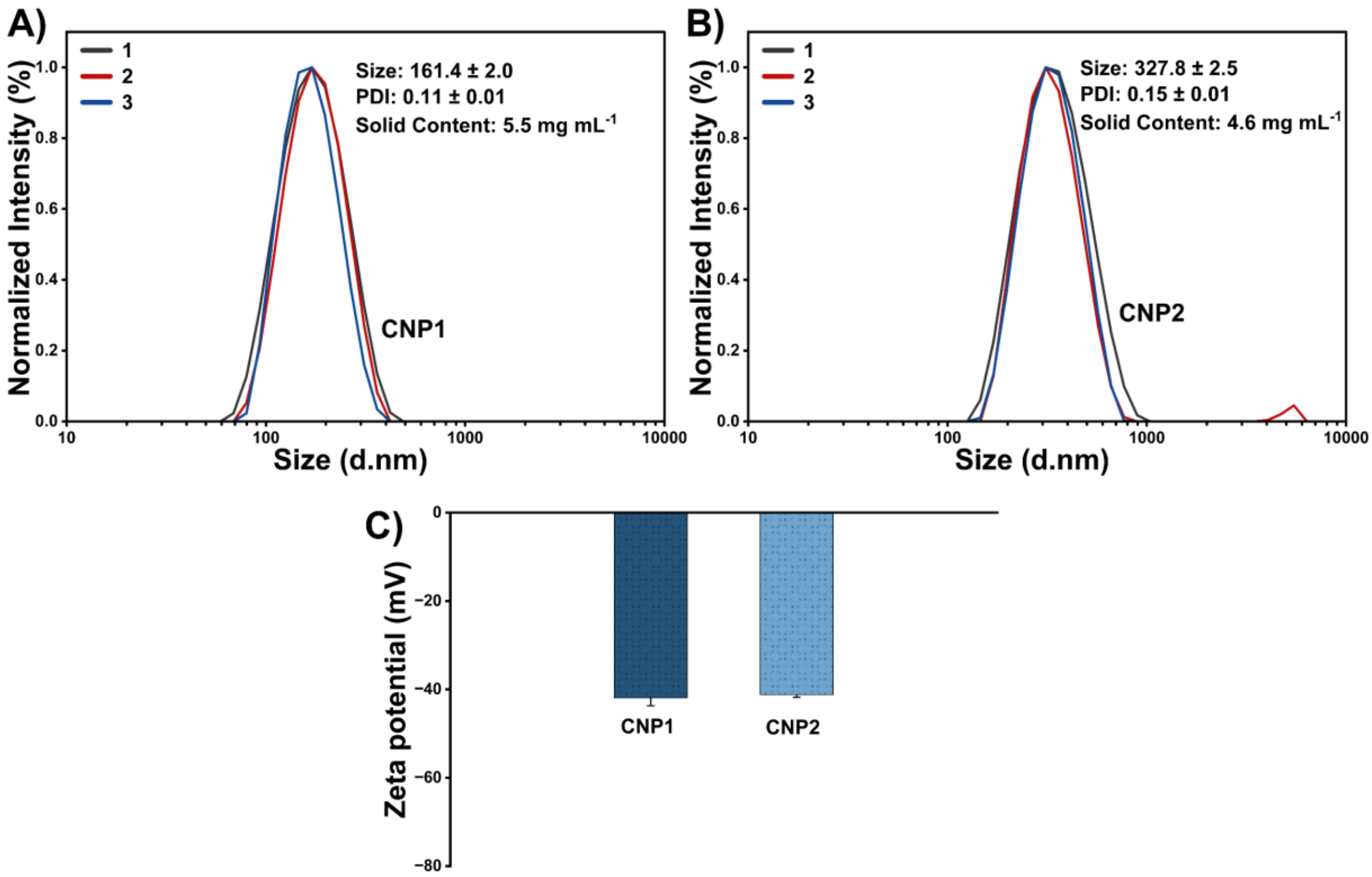

Figure S7. Particle size comparison of CNPs in the presence of surfactants (TW20) was used as a control experiment to evaluate the effect of surfactant on particle size distribution. **A)** Size distribution profile of CNP1 (containing 1% TW20), **B)** CNP2 (containing 0.1% TW20 as an equivalent amount for the shell structure of Maillard complex, **C)** zeta potential (mV) values of these CNPs

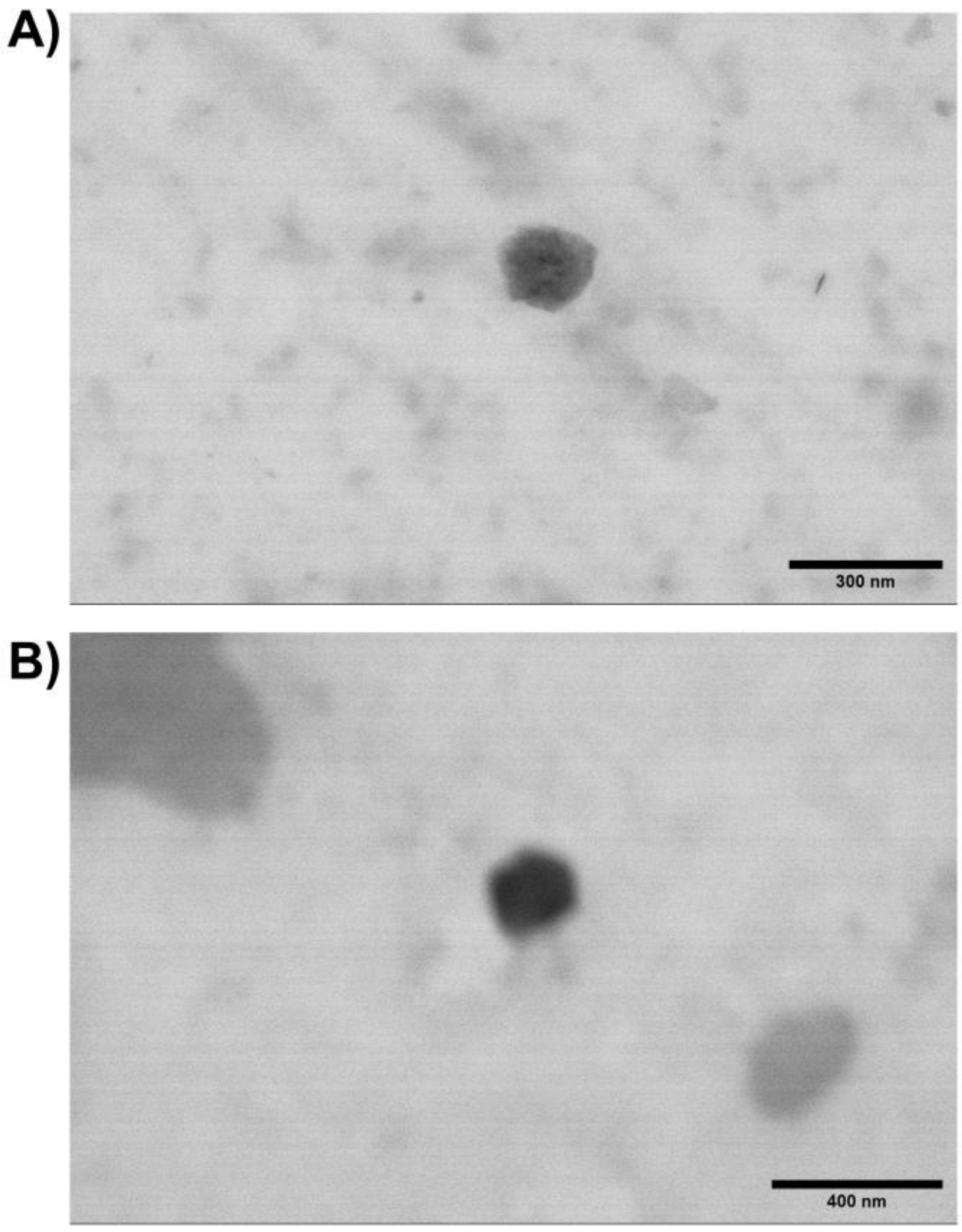

Figure S8. STEM (Scanning Transmission Electron Microscopy) images of control nanoparticle formulations: **A)** CNP1 (containing 1% (v/v) TW20), and **B)** CNP2 (containing 0.1% (v/v) Tween 20, corresponding to the equivalent biopolymer shell concentration)

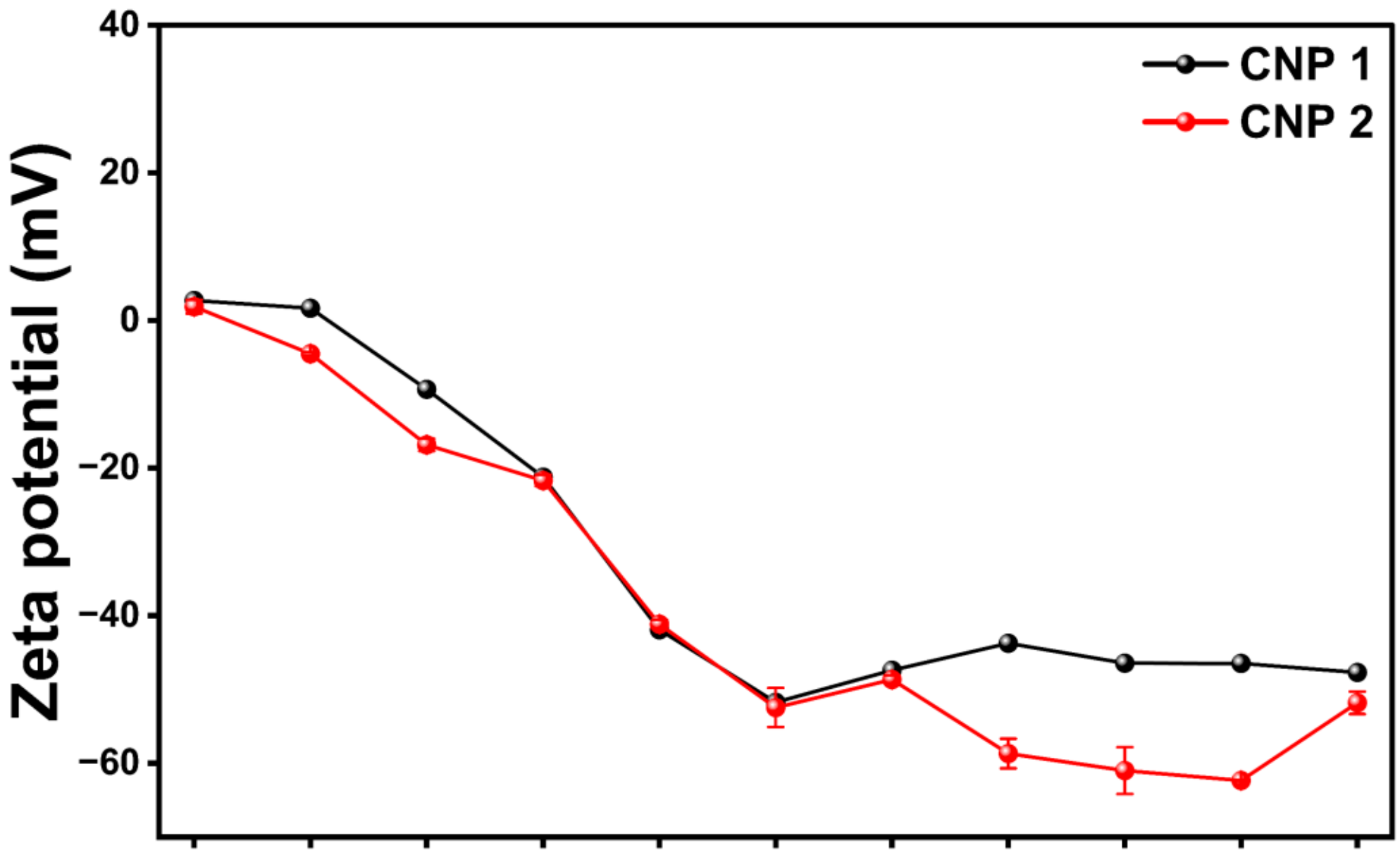

Figure S9. pH-dependent zeta potential of CNP1 and CNP2 samples

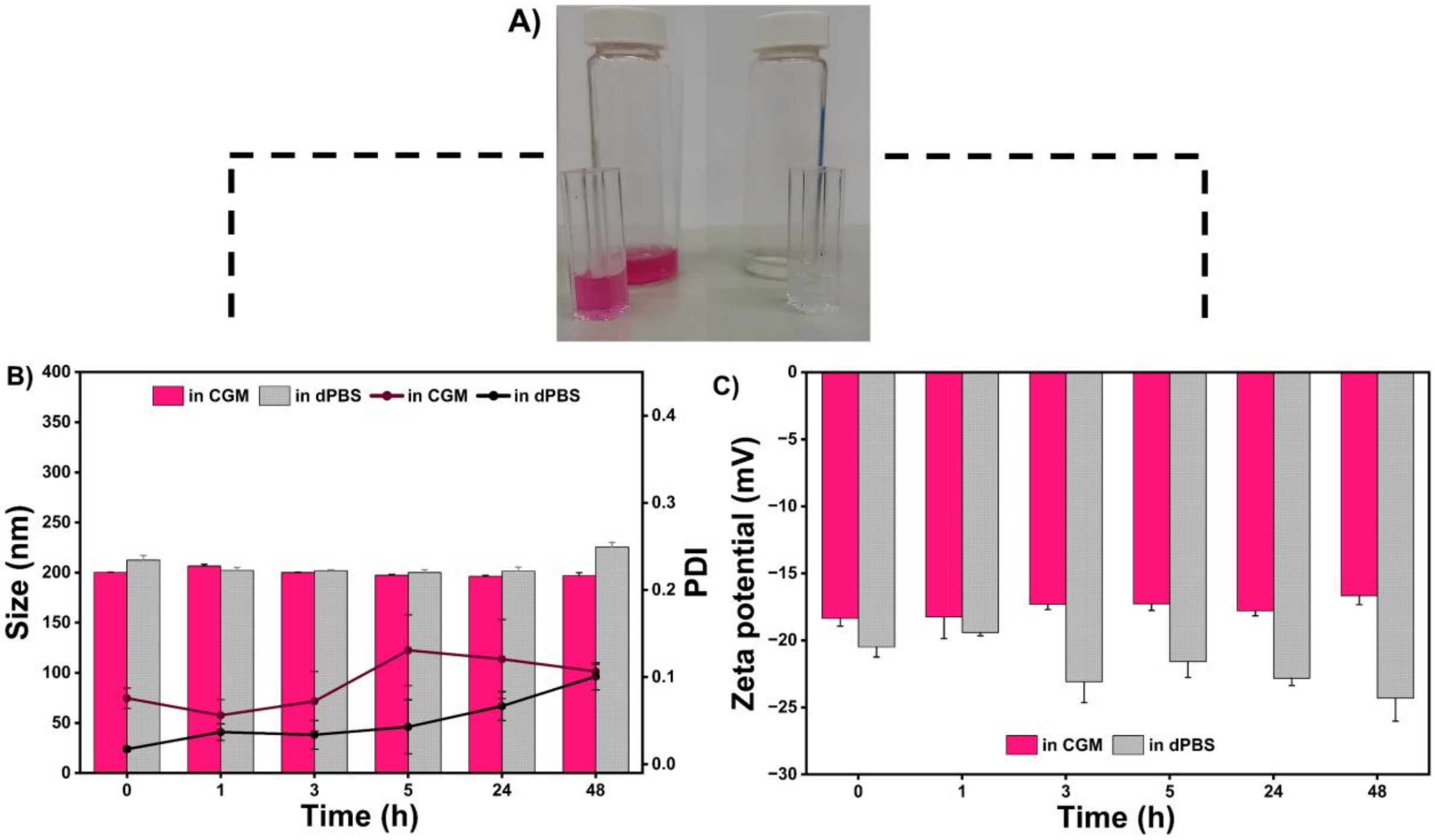

Figure S10. Colloidal stability of HNP incubated at 37 °C in PBS and complete growth medium (CGM) over the experimental time period, **A)** experimental setup for measurements, **B)** evaluated hydrodynamic size (nm) and PDI, **C)** zeta potential (mV) values

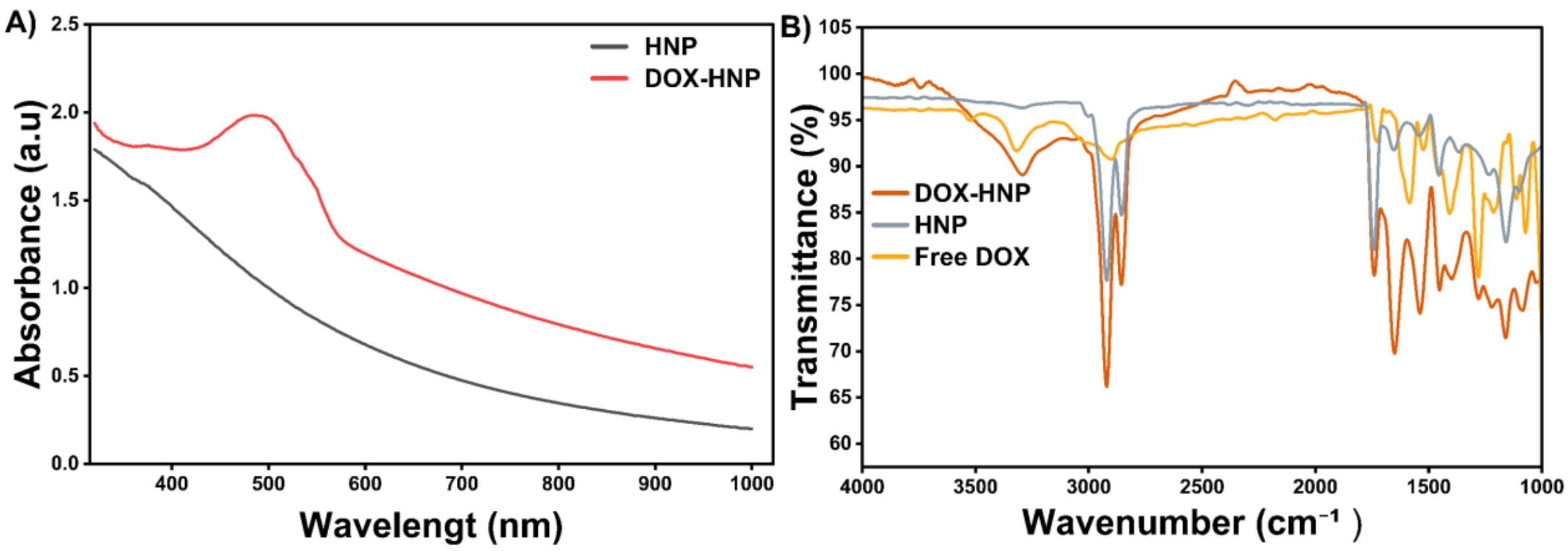

Figure S11. A) UV-Vis absorbance spectrum of HNP and 5 mg loaded DOX-HNP **B)** FT-IR spectrum of free DOX

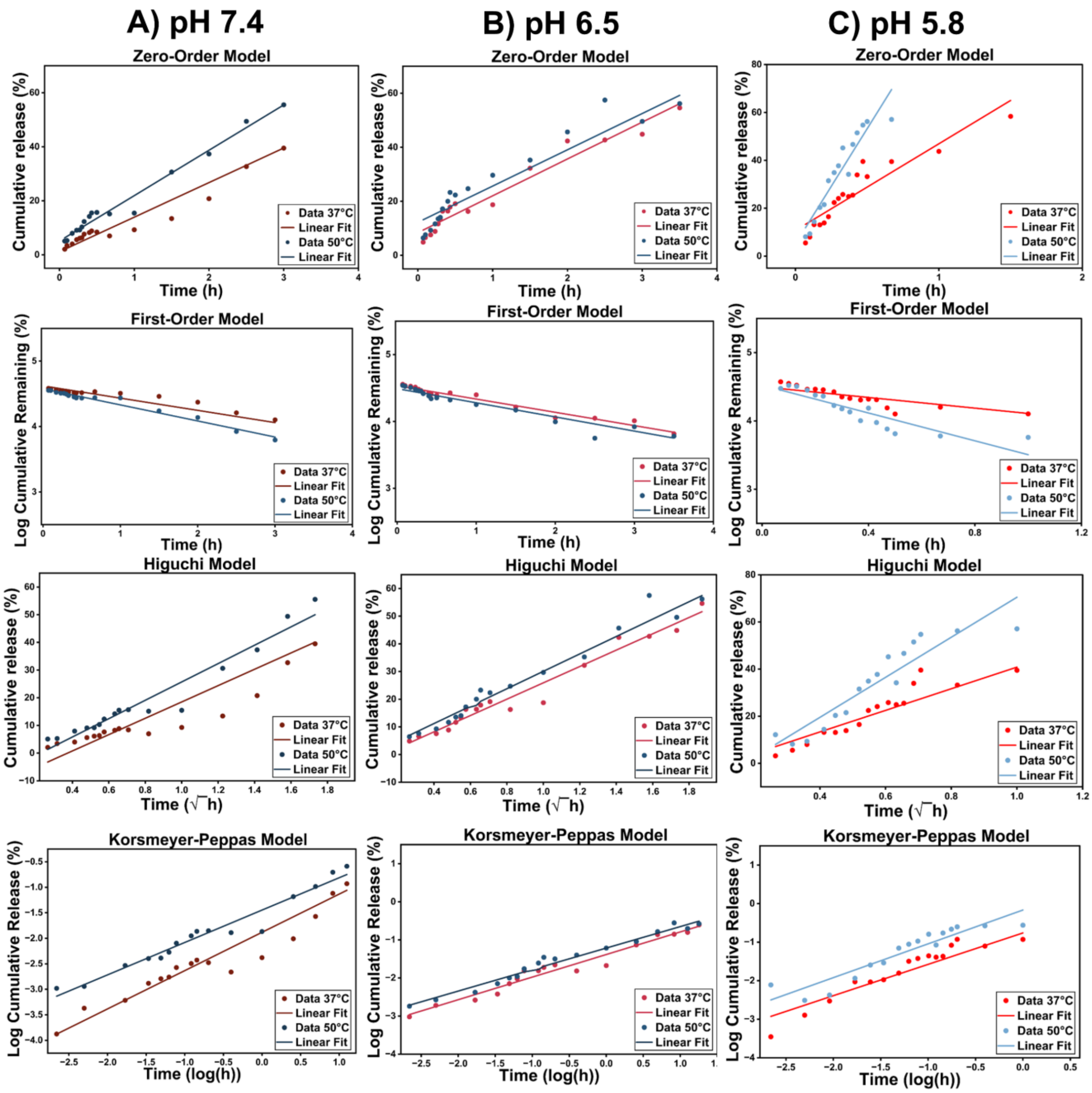

Figure S12. Kinetic *in vitro* drug release models of DOX from DOX-HNP under different pH conditions and temperatures. **A)** Release at PBS (pH 7.4), **B)** KPHO (pH 6.5), and **C)** KPHO, pH 5.8. For each pH condition, drug release profiles were measured at 37°C and 50°C. Data used for model fitting were limited to the **first 60% of cumulative release** to ensure accuracy of the kinetic parameters.

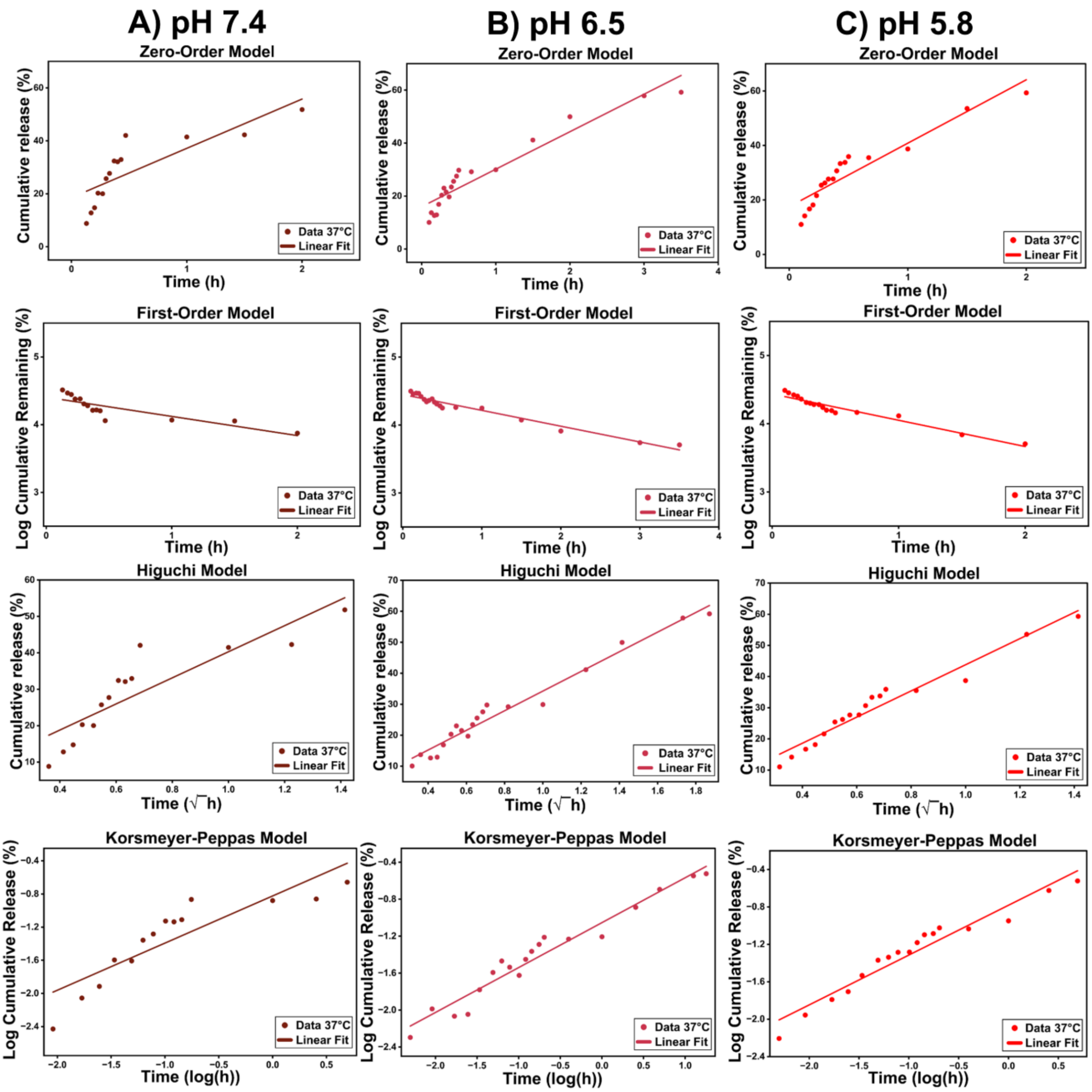

Figure S13. Kinetic *in vitro* drug release models of DOX from DOX-HNP with Proteinase K enzyme. **A)** Release at PBS (pH 7.4), **(B)** KPHO (pH 6.5), and **(C)** KPHO (pH 5.8). For each pH condition, drug release profiles were measured at 37°C. Data used for model fitting were limited to the **first 60% of cumulative release** to ensure accuracy of the kinetic parameters.

Table S5. Kinetic Model Parameters (n) and Correlation Coefficients (R^2) Calculated from the Initial Release Phase (≤60% Cumulative Release) of DOX from DOX-HNP at pH 7.4, 6.5, and 5.8 at 37°C and 50°C (with and without enzyme).

Release Conditions	Enzyme	Temperature	Zero-order model	First-order model	Higuchi model	Korsmeyer-Peppas model	
			R^2	R^2	R^2	R^2	n
**pH 7.4**	-	37°C	0.97	0.96	0.91	0.94	0.75
		50°C	0.98	0.97	0.94	0.96	0.63
	+	37°C	0.66	0.73	0.77	0.78	0.57
**pH 6.5**	-	37°C	0.95	0.97	0.97	0.96	0.59
		50°C	0.91	0.93	0.97	0.98	0.56
	+	37°C	0.91	0.95	0.97	0.95	0.48
**pH 5.8**	-	37°C	0.84	0.86	0.90	0.91	0.51
		50°C	0.65	0.72	0.80	0.84	0.56
	+	37°C	0.87	0.93	0.95	0.94	0.53

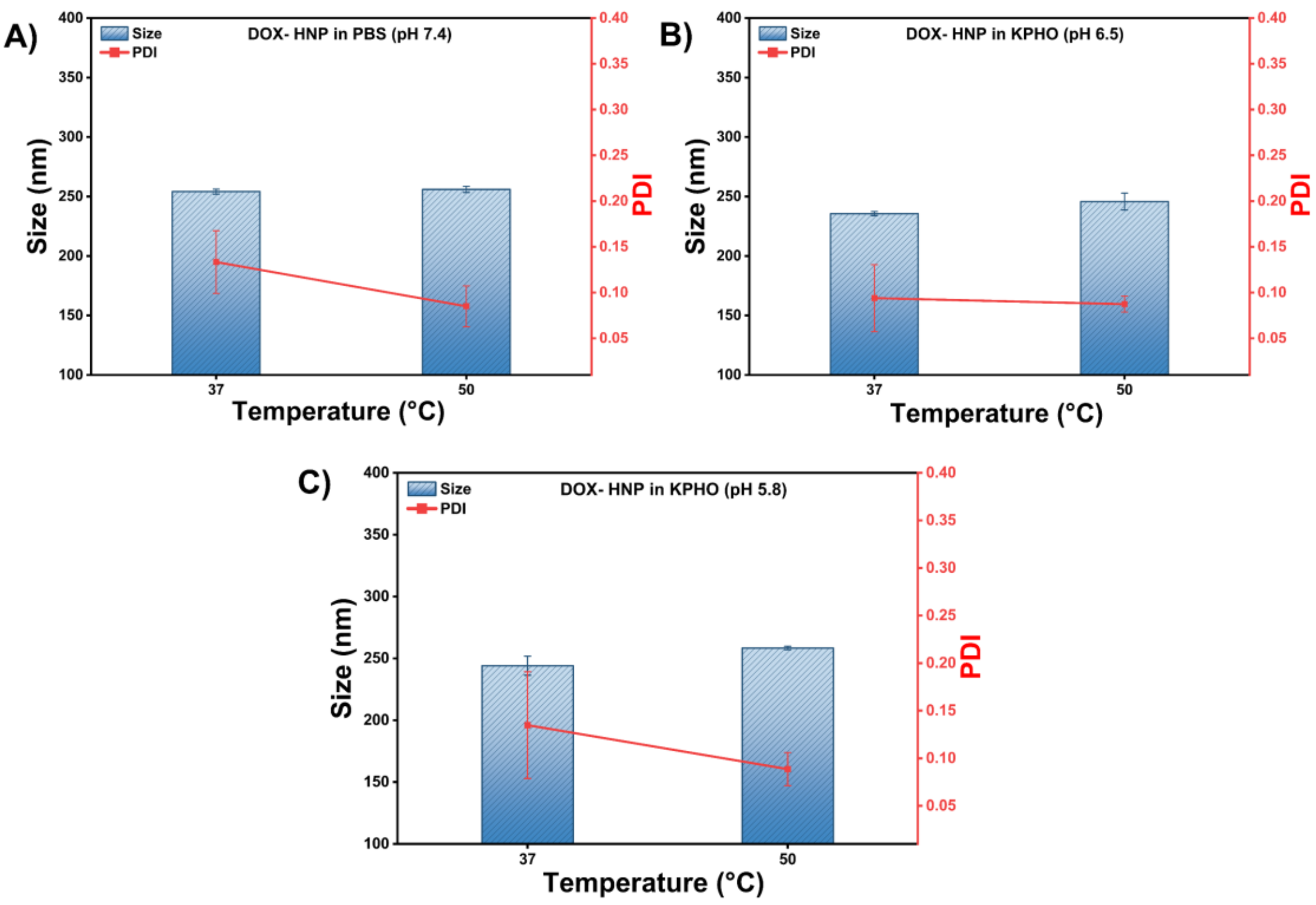

Figure S14. Comparison of sample sizes obtained from the membrane after release at 37°C and 50°C. Samples were collected through the membrane after 96 h and analyzed to assess the effect of temperature on particle size

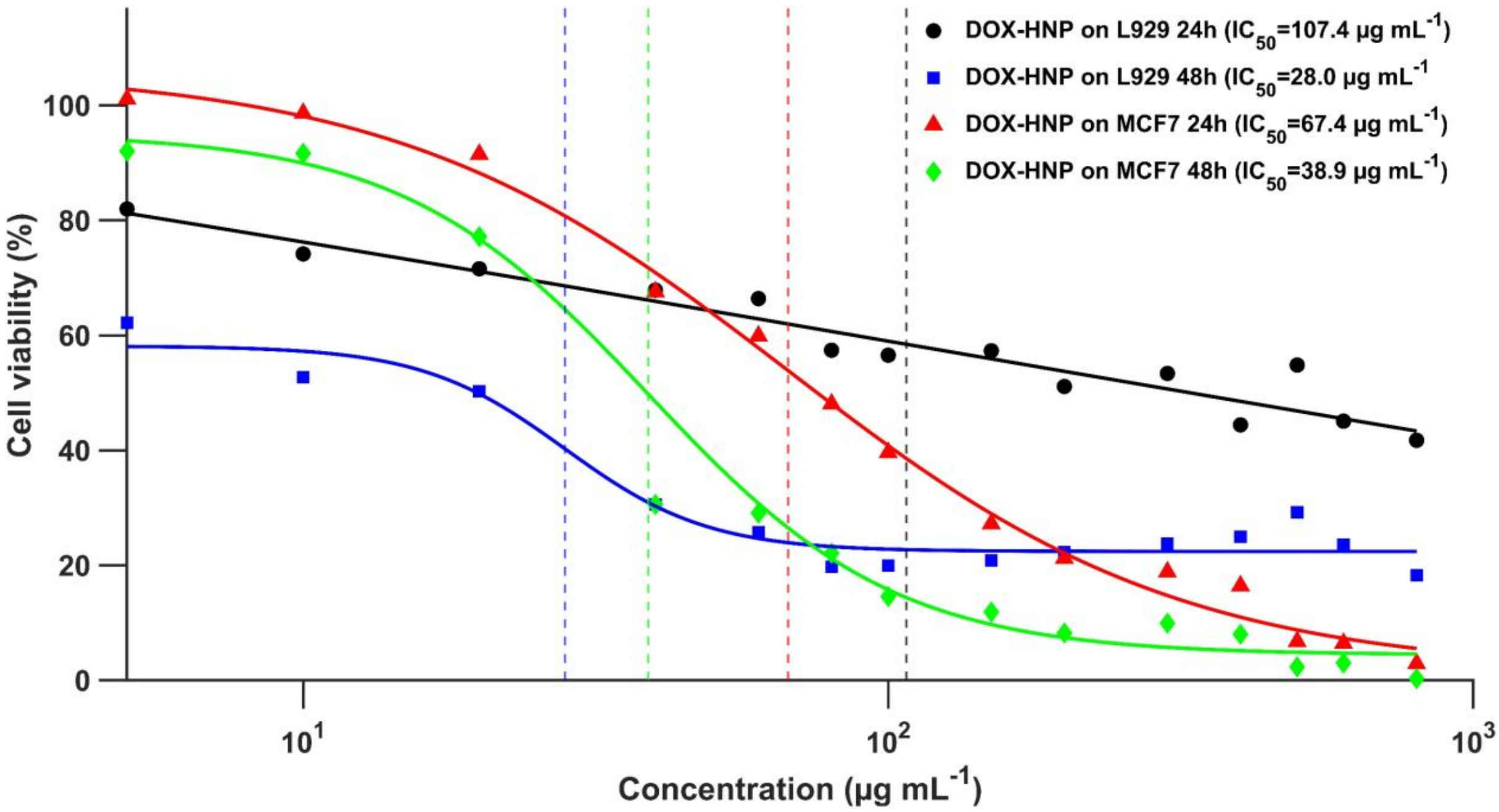

Figure S15. IC_{50} analysis of DOX-HNP formulations on L929 and MCF7 cell lines after 24 h and 48 h incubation periods. Nonlinear regression analysis was performed using a four-parameter logistic model to determine IC_{50} values.

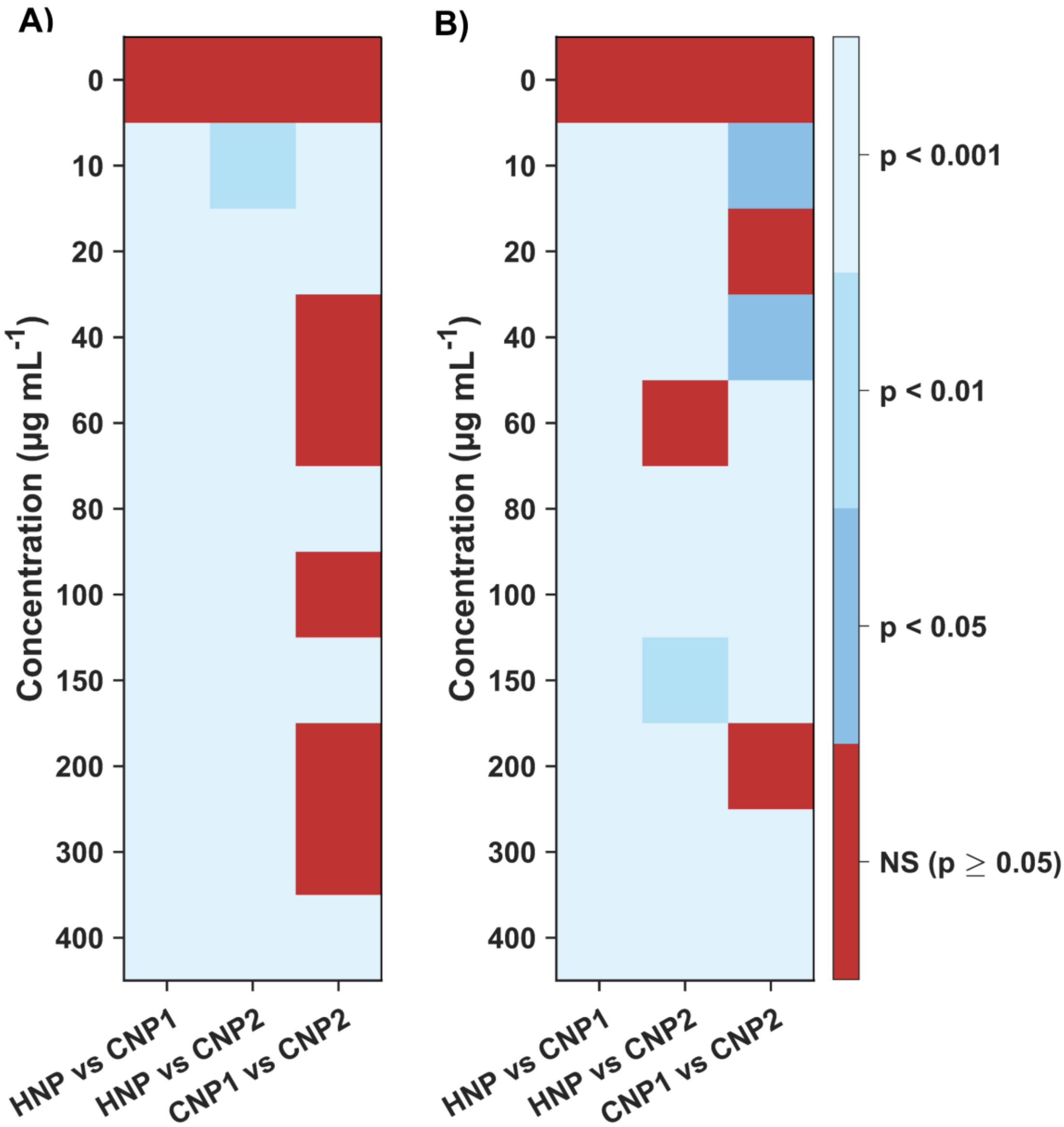

Figure S16. Statistical analysis of MTT assay in L929 cells. Two-way ANOVA followed by Tukey's post hoc test was used to assess differences among HNP, CNP1 (1% (v/v) TW20 NP), and CNP2 (1 mg mL^{-1} TW20 NP) at **A)** 24 h, and **B)** 48h across all concentrations (n = 3).
